# Optimal exercise dose for resistance training to improve HbA1c levels in overweight and obese patients with type 2 diabetes: a systematic review of randomized controlled trials and a Bayesian network meta-analysis

**DOI:** 10.3389/fphys.2026.1867073

**Published:** 2026-08-12

**Authors:** Huarui Li, Shidong Yang, Xuefeng Wang, Zhiwei Wang, Wei Zhang, Xuehai Zhang, Shouliang Huang

**Affiliations:** 1 School of Physical Education, Nanjing XiaoZhuang Unversity, Nanjing, China; 2 College of Physisal Education and Health, Guangxi Normal University, Guilin, China

**Keywords:** HbA1c, obesity, optimal dose, over-weight, resistance training, type 2 diabetes

## Abstract

**Background:**

The high prevalence of overweight or obesity in patients with type 2 diabetes (T2DM) is strongly associated with impaired quality of life and an increased risk of long-term complications. Poor glycemic control remains a fundamental clinical challenge in this population. Although resistance training (RT) is an effective intervention for improving HbA1c levels, the optimal dosing parameters​ (e.g., frequency, intensity, volume) have not been systematically established. Therefore, this meta-analysis aimed to investigate the dose-response relationships​ between various RT regimens and HbA1c reduction in overweight or obese individuals with T2DM.

**Methods:**

This systematic review and network meta-analysis, conducted by searching PubMed, Embase, and the Cochrane Library, included randomized controlled trials on resistance training in patients with T2DM who were overweight or obese, conducted from the inception of the databases through January 19, 2026. Comprehensive data extraction covered training dose, resistance training protocols, participants’ demographic characteristics, and study duration.

**Results:**

A total of 10 studies involving 633 participants (mean age: 60.03 ± 7.77 years; mean BMI: 30.69 ± 6.64; Male: 32.7%) were included in this study. The results of our network meta-analysis showed that resistance training variables (e.g., frequency, intensity, period, and training volume) were effective in improving HbA1c in overweight or obese patients with T2DM. Among them, the effective dose range for frequency of resistance training was 3 times/week, resistance training intensity was 30-62%, resistance training period was 17–23 weeks, resistance training time was 45 minutes per session, resistance training exercises was 10 exercises per set, resistance training repetitions was 13–18 reps, resistance training sets was 2–4 sets, resistance training volume was 1000–2100 reps per week. the analysis suggested a potential optimum around of resistance training to improve HbA1c in overweight or obese patients with T2DM is 3 times/week (MD=-0.35, 95% CrI [-0.62, -0.08]), 44% 1RM (MD=-0.41, 95% CrI [-0.74, -0.08]), 23 weeks (MD = −0.35, 95% CrI [−0.68, −0.02]), 45min (MD = −0.32, 95% CrI [−0.62, −0.01]), 10 exercises (MD = −0.32, 95% CrI [−0.63, −0.01]), 18 reps (MD=-0.38; 95% CrI [-0.74, -0.02]), 3 sets (MD=−0.36, 95% CrI [−0.63, −0.10]), 1,500 reps/week (MD = −0.51, 95% CrI [−0.86, −0.15]).

**Conclusion:**

Resistance training effectively improvesHbA1c in overweight or obese patients with T2DM. A recommended 23-week program includes 3 sessions per week at 44% 1RM, 45min per session, featuring 10 exercises per set, 3 sets, and 18 repetitions per exercise, totaling up to 1500 reps weekly.

**Systematic Review Registration:**

https://www.crd.york.ac.uk/prospero/, identifier CRD420261341070.

## 1 Introduction

Type 2 diabetes mellitus (T2DM) has become a major global public health challenge.​ According to the International Diabetes Federation, the number of individuals with diabetes continues to rise, with Type 2 diabetes accounting for the vast majority of cases (Collaborators G B D D, 2023). Overweight and obesity are prominent risk factors for T2DM and frequently coexist, creating a deleterious cycle of metabolic dysregulation that amplifies the risk of complications, including cardiovascular and renal disease (Lean et al., 2018). Glycated hemoglobin (HbA1c) is the gold standard for assessing long-term glycemic control in patients with diabetes (World Health Organization, 2011; Sherwani et al., 2016).Elevated HbA1c correlates strongly with both microvascular and macrovascular complications (American Diabetes Association Professional Practice C, 2024), therefore, identifying effective intervention strategies to improve HbA1c levels is of critical importance.

Exercise interventions constitute a cornerstone of diabetes management. Although aerobic exercise has traditionally been regarded as the preferred modality for improving glycemic control (Colberg et al., 2016). the role of resistance training (RT) has gained increasing recognition. Skeletal muscle is the primary site for glucose uptake and utilization, accounting for approximately 80% of postprandial glucose disposal (Petersen and Shulman, 2018). Resistance training uniquely enhances insulin sensitivity by increasing skeletal muscle mass, optimizing muscle fiber composition, upregulating insulin receptor expression, and promoting the translocation of glucose transporter 4 (GLUT4) (Holten et al., 2004). Numerous systematic reviews and meta-analyses have demonstrated that RT significantly reduces HbA1c in patients with T2DM, with weighted mean differences ranging from approximately −0.30% to −0.39% (Jansson et al., 2022). Notably, improvements in muscular strength correlate positively with HbA1c reductions.

Against this mechanistic backdrop, recent large-scale network meta-analyses have begun to clarify which types of exercise​ most effectively modify metabolic outcomes and body composition in diabetes-related populations. Khalafi et al. (2026a) reported on the comparative efficacy of exercise modalities for reducing visceral adipose tissue in prediabetes and T2DM, while Khalafi et al. (2026b)​ examined effects on intrahepatic lipid content, glucose homeostasis, and liver function—findings that underscore the metabolic relevance of exercise modality selection (Khalafi et al., 2026). Similar systematic efforts have also characterized exercise effects on body composition and cardiometabolic risk factors in type 1 diabetes (Khalafi et al., 2025), highlighting the broader therapeutic potential of structured training across diabetes phenotypes. However, these studies predominantly compare exercise types (aerobic, combined, HIIT, resistance) rather than dissect the internal dose–response structure of resistance training itself. Existing systematic reviews in T2DM often pool heterogeneous populations or report only summary effects without quantifying how specific RT parameters—intensity, volume, frequency, sets, repetitions—jointly determine HbA1c reduction. This limitation is particularly salient for overweight or obese patients, where exercise capacity and adherence may shift the effective dose range, yet precise, individualized RT prescriptions remain lacking.

Therefore, this study aims to systematically review the evidence regarding the effects of resistance training on HbA1c levels in overweight or obese patients with T2DM. It seeks to explore the optimal combination of multidimensional parameters—including frequency, intensity, frequency, duration, number of exercises, repetitions, and exercise volume—to provide evidence-based support for the development of scientifically sound, safe, and effective resistance training programs. This effort will contribute to the precision and standardization of exercise prescriptions for diabetes management.

## 2 Methods

This pre-registered systematic review with meta-analysis (PROSPERO reference number #CRD420261341070) was reported following the PRISMA checklist (Moher et al., 2009; Page et al., 2021).

### 2.1 Search strategy

We systematically searched PubMed, Embase, and the Cochrane Central Register of Controlled Trials (CENTRAL) from inception to January 19, 2026. The search strategy combined Medical Subject Headings (MeSH) and free-text terms related to: (1) resistance training (e.g., “resistance training,” “strength training,” “weight lifting”); (2) type 2 diabetes (e.g., “diabetes mellitus, type 2,” “T2DM”); (3) overweight and obesity (e.g., “overweight,” “obesity,” “body mass index”); and (4) HbA1c (e.g., “glycated hemoglobin,” “HbA1c”). The complete electronic search syntax for each database is provided in **Supplementary File 1**.

**Figure 1:**
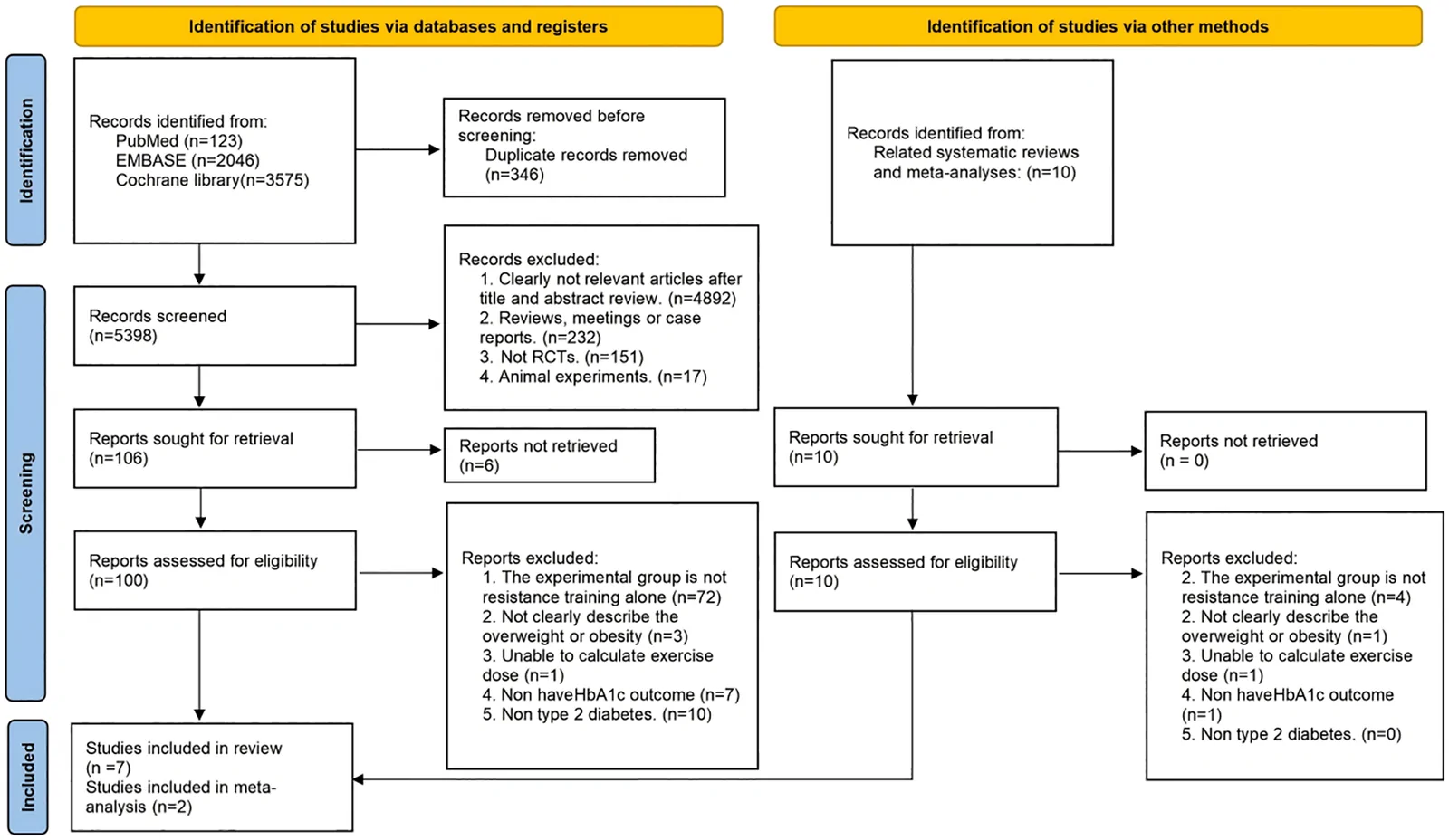
PRISMA Flow diagram of the search process for studies.

Reference lists of relevant reviews and meta-analyses were manually screened to identify additional eligible studies. No language restrictions were applied beyond the requirement for English-language full texts. Gray literature sources (e.g., trial registries, dissertations, and conference abstracts) were not systematically searched. Title and abstract screening, followed by full-text screening, were conducted independently and in duplicate by two investigators (L.H.R. and Y.S.D.). Inter-rater reliability was quantified using Cohen’s kappa statistic, which indicated excellent agreement beyond chance (title/abstract: κ = 0.86, 95% CrI [0.78–0.94]; full text: κ = 0.91, 95% CrI [0.84–0.98]). Disagreements were resolved by discussion or adjudication by a third author (W.X.F.). The study selection process is summarized in a PRISMA flow diagram (**Figure 1**).

### 2.2 Selection criteria

We included (1) randomized controlled trials in which (2) participants were assessed and had available data indicating the presence of type 2 diabetes, and (3) overweight or obesity, (4) used resistance training as the intervention, and (5) the control group (CON) consisted of no intervention, usual daily activities, health education, or standard care. To be included, the study must also have included glycated hemoglobin as an outcome measure for type 2 diabetes. We excluded (1) non-English publications; (2) studies that did not clearly describe the dose of resistance training; and (3) duplicate publications.

Definition of overweight and obesity: Overweight and obesity were defined using two complementary criteria: (i) the World Health Organization (WHO) international classification: overweight as body mass index (BMI) ≥25 kg/m² and obesity as BMI ≥30 kg/m²; and (ii) the WHO Western Pacific Regional Office (WPRO) criteria for Asian populations: overweight as BMI ≥23 kg/m² and obesity as BMI ≥25 kg/m² (Consultation W H O E, 2004). Studies were eligible if participants met either definition. When studies reported only mean BMI values (e.g., ~26 kg/m²), we considered participants eligible if these values met the relevant cutoff. Upper BMI limits applied within individual RCTs (e.g., BMI <35 kg/m²) were not used as inclusion criteria for overweight/obesity in this review but are reported descriptively.

### 2.3 Data extraction

Two authors independently extracted data from studies that met the inclusion criteria (LHR and HSL) and disagreements were resolved by consensus between all authors. From each of the included studies, we extracted relevant publication information (i.e., author and year), number of patients, patient characteristics (e.g., age and sex), interventions considered and outcome measures (HbA1c). In the process of extracting data, if the original study reported a standard error in the experimental and control groups, the standard deviation was calculated by the formula: In cases where both standard deviation (SD) and other direct measures are unavailable, we will derive the SD using methods based on credible intervals, quartiles, ranges, or p-values. These methods are outlined in detail in Sect. 7.7.3 of the Cochrane Handbook for Systematic Reviews of Interventions.

### 2.4 Data coding and management

We categorized the interventions into two hierarchical levels: First, interventions were coded as “RT” or “CON” (first level). At second level, the interventions were coded according to resistance training variables: Frequency (number of resistance training sessions completed per week, 2–3 sessions/week), Intensity (training load of a single resistance training session, 25%-80% 1RM), Period (duration of resistance training, 12–24 weeks), Exercises (number of exercises per session, 5–10 exercises), Sets (number of sets completed per resistance training session, 2.5–4 sets), Repetitions (number of repetitions of individual exercise components, 7–19 reps), Training volume (total amount of training per week, derived from the product of frequency, exercises, repetitions, and sets, 200–2280 reps/week) and Time (duration of each resistance training session, 40–60 min). Although training volume can be derived as the product of frequency, exercises, sets, and repetitions, it was treated as an independent coded variable in the MBNMA to capture potential nonlinear cumulative effects; thus, the optimal volume estimate may slightly differ from the arithmetic product of the optimal constituent parameters. In order to avoid bias of recorded information caused by subjective factors, the following recording methods were adopted for each element of exercise doses: (1) The original values were maintained for the literature with fixed dose values such as exercise intensity, frequency and times; (2) When the dose of exercise intensity, frequency and time is in a certain range, the average value of the range is taken; (3) The exercise intensity included in this study was varied such as Borg rating of perceived exertion (Borg RPE15), Borg category-ratio scale (Borg CR10), etc. To enable dose–response modeling across studies using heterogeneous reporting formats, one-repetition maximum (1RM) was adopted as the uniform metric for resistance training intensity. For studies reporting 1RM directly, original values were retained. For studies reporting intensity using the Borg Rating of Perceived Exertion (RPE), RPE scores were converted to approximate 1RM percentages based on established psychophysical relationships for resistance training prescription (Borg, 1982; Higgins et al., 2012). This approach is consistent with standardized practices in exercise science and allowed for meaningful comparisons of intensity across studies. Other resistance training variables—including frequency, period, number of exercises, repetitions, sets, and session duration—were coded using original reported values or midpoint estimates for ranges, as appropriate. The final analytical dataset is provided in **Supplementary File 6**.

### 2.5 Data synthesis

We conducted a random-effects Bayesian Model-Based Network Meta-Analysis (MBNMA) (Mawdsley et al., 2016) to estimate the dose–response relationship between resistance training variables and HbA1c in overweight and obese patients with type 2 diabetes, using R statistical environment (version 4.4.1; R Foundation for Statistical Computing, Vienna, Austria; www.r-project.org). Key assumptions for network meta-analysis—including network connectedness (Ter Veer et al., 2019), consistency, and transitivity (Wheeler et al., 2010; White et al., 2012)—were verified and showed no violations (**Supplementary File 4**). Effect sizes are reported as mean differences (MD) with 95% credible intervals (CrI) (Etzioni and Kadane, 1995). Multi-arm trials with multiple resistance training doses versus a shared control were accommodated within the MBNMA framework by modeling a common study-level random effect across all arms, thereby preserving the within-study correlation structure.

Initial visual inspection of the observed dose–response relationships informed the selection of candidate non-linear functions, including Emax, quadratic, non-monotonically up, and restricted cubic splines (RCS) (Pedder et al., 2019). Model fit was compared across these candidates using goodness-of-fit indices: Deviance Information Criterion (DIC), residual deviance, between-study standard deviation, number of model parameters, and corresponding deviance plots (Evans, 2019) (**Supplementary File 5**). In the final RCS models, knots were placed at the 10th, 50th, and 90th percentiles of the dose distribution for each resistance training variable, balancing model flexibility with biological plausibility (Pedder et al., 2019). Departure from linearity was assessed using a Wald test (Nunez et al., 2011; Hamza et al., 2021). Beta coefficients from the RCS models were used to estimate the predicted dose at which the maximal significant effect on HbA1c was achieved. The flowchart for the network meta-analysis is provided in **Supplementary File 3**.

## 3 Results

### 3.1 Characteristics of included studies

Overall, 5744 records were identified through the initial electronic searches. After removing duplicates, 5398 records were screened for titles and abstracts and 106 full-text articles were screened for eligibility. A total of 10 records that may have been missed during the search were obtained by reviewing recent reviews or similar meta-analyses related to the search topic and 10 full-text articles were screened for eligibility. In total, 10 studies involving 633 participants (332 treatments and 301 controls) were included in the review (**Figure 1**).

The characteristics of the included studies are shown in **Table 1**. The year of publication ranged from 2002 and 2024. A total of 207 (32.7%) participants were men, Of these, 69 did not report their gender (10.9%). We also reported on the tools used in the study to assess type 2 diabetes and their diagnostic thresholds. Several studies used HbA1c levels between 6.5% and 11.0% as one of the inclusion criteria for type 2 diabetes (5 studies), 2 studies did not specify the assessment tools or diagnostic thresholds for type 2 diabetes, and 3 studies used different diagnostic criteria and their respective cutoff values.

**Table 1:** Basic characteristics of included studies.

Study ID	Age (mean ± SD)	Sample size (M/F)	HbA1c (%)	BMI	Diagnosis of type 2 diabetes	Intervention detail
Mondal et al. (2021)	40-64	CON: 6(-/-) RT: 6(-/-)	CON: 8.8 ± 2.1 RT: 8.7 ± 1.5	BMI<35kg/m^2^	HbA1c levels between 6.5% and 11.0%	RT: 16weeks, 40 min, 3 times/week, 6 exercises, 70%-80% 1RM, 8–11 reps/set, 3–4 sets.
Ma et al. (2024)	CON: 56.29 ± 5.92 RT: 57.56 ± 4.85	CON: 32(16/16) RT: 31(11/20)	CON: 8.23 ± 1.02 RT: 8.01 ± 0.83	CON: 26.92 ± 1.74 RT: 26.33 ± 1.76	HbA1c levels between 6.5% and 11.0%	RT: 12weeks, 40 min, 3 times/week, 9 exercises, 20%-40% 1RM, 15–30 reps/set, 4 sets.
Ma et al. (2024)	CO: 65.55 ± 4.41 RT: 66.65 ± 4.94	CON: 33(14/19) RT: 31(13/18)	CON: 7.98 ± 0.96 RT:7.80 ± 0.93	CON: 26.56 ± 1.58 RT: 26.02 ± 2.05	HbA1c levels between 6.5% and 11.0%	RT: 12weeks, 40min, 3 times/week, 10 exercises, 40%-60% 1RM, 8–15 reps/set, 2–4 sets.
Ma et al. (2024)	CON: 65.55 ± 4.41 RT: 66.41 ± 4.97	CON: 33(14/19) RT: 34 (12/22)	CON: 7.98 ± 0.96 RT: 7.75 ± 0.97	CON: 26.56 ± 1.58 RT: 26.31 ± 1.88	HbA1c levels between 6.5% and 11.0%	RT: 24weeks, 40min, 3 times/week, 10 exercises, 20%-30% 1RM, 15–30 reps/set, 2–4 sets.
Kadoglou et al. (2012)	CON:64.6 ± 4.3 RT:61.5 ± 5.4	CON: 24(5/19) RT: 23(7/16)	CON:7.5 ± 0.5 RT:7.4 ± 0.4	CON:31.58 ± 5.71 RT:32.74 ± 4.05	HbA1c levels >6.5%	RT: 12weeks, 45 min-60 min, 3 times/week, 8 exercises, 60%-80% 1RM, 6–8 reps/set, 2-3sets.
Giessing et al. (2022)	CON:64 ± 7 RT:61 ± 5	CON: 28(-/-) RT: 29(-/-)	CON:8.3 ± 0.7 RT:8.4 ± 0.9	CON:31.7 ± 5.3 RT:32.1 ± 6.3	HbA1c level ≥6.3%; FBG≥100mg/dl	RT: 24weeks, 45 min, 2 times/week, 10 exercises, 67%-80% 1RM, 8–12 reps/set, 1 set.
Castaneda et al. (2002)	CON: 66 ± 1 RT: 66 ± 2	CON: 31(12/19) RT: 31(10/21)	CON: 8.4 ± 0.3 RT: 8.7 ± 0.3	CON: 31.2 ± 1.0 RT: 30.9 ± 1.1	FBG≥7.0 mmol/l	RT: 16weeks, 45 min, 3 times/week, 6 exercises, 60%-80% 1RM, 8 reps/set, 3 sets.
Baldi and Snowling, (2003)	CON: 50.1 ± 1.3 RT: 46.5 ± 2.1	CON: 9(9/0) RT: 9(9/0)	CON: 8.5 ± 0.8 RT: 8.9 ± 1.2	CON: 36.4 ± 3.1 RT: 34.3 ± 3.2	NA	RT: 10weeks, 40 min, 3 times/week, 10 exercises, 70% 1RM, 12 reps/set, 2 sets.
Sigal et al. (2007)	CON:54.8 ± 7.2 RT:54.7 ± 7.5	CON: 63(41/22) RT: 64(40/24)	CON:7.66 ± 0.89 RT: 7.71 ± 0.86	CON: 35.0 ± 9.5 RT: 34.1 ± 9.6	HbA1c levels between 6.6% and 9.9%. American Diabetes Association	RT: 22weeks, 45 min, 3 times/week, 7 exercises, 80% 1RM, 8 reps/set, 2–3 sets.
Jorge et al. (2011)	CON:53.42 ± 9.82 RT:54.10 ± 8.94	CON: 12(4/8) RT: 10(4/6)	CON:7.04 ± 0.68 RT: 8.51 ± 2.46	CON: 30.03 ± 4.90 RT: 31.29 ± 4.08	NA	RT: 12weeks, 60min, 3 times/week, 7 exercises, 50% 1RM, 8–12 reps/set, 4 sets.
Cheung et al. (2009)	CON:65.55 ± 4.41 RT:66.65 ± 4.94	CON: 63(41/22) RT: 64(40/24)	CON:7.4 ± 1.0 RT: 7.2 ± 1.6	CON: 37.7 ± 9.2 RT: 39.7 ± 9.0	NA	RT: 16weeks, 40 min, 3 times/week, 7 exercises, 67% 1RM, 12 reps/set, 2 sets.

M/F, Male or Female; RT, Resistance training; CON, control group; BMI, Body Mass Index; Overweight and obesity were defined according to WHO international criteria (overweight: BMI ≥25 kg/m²; obesity: BMI ≥30 kg/m²) and WHO Western Pacific Regional Office (WPRO) criteria for Asian populations (overweight: BMI ≥23 kg/m²; obesity: BMI ≥25 kg/m²). The BMI <35 kg/m² reported in Soumitra Mondal et al. (2021) reflects an upper eligibility limit within that trial, not the definition of overweight/obesity used in this review; NA: Not available Two rows correspond to distinct resistance training arms (differing in dose parameters) from the same multi-arm RCT by Ma et al. (2024), sharing a single control group. Clustering within this trial was accounted for via the hierarchical structure of the Bayesian Model-Based Network Meta-Analysis (MBNMA).3.2 Dose-response relationship.

#### 3.2.1 The effect of resistance training frequency on HbA1c levels in overweight/obese patients with T2DM

**Figure 2:**
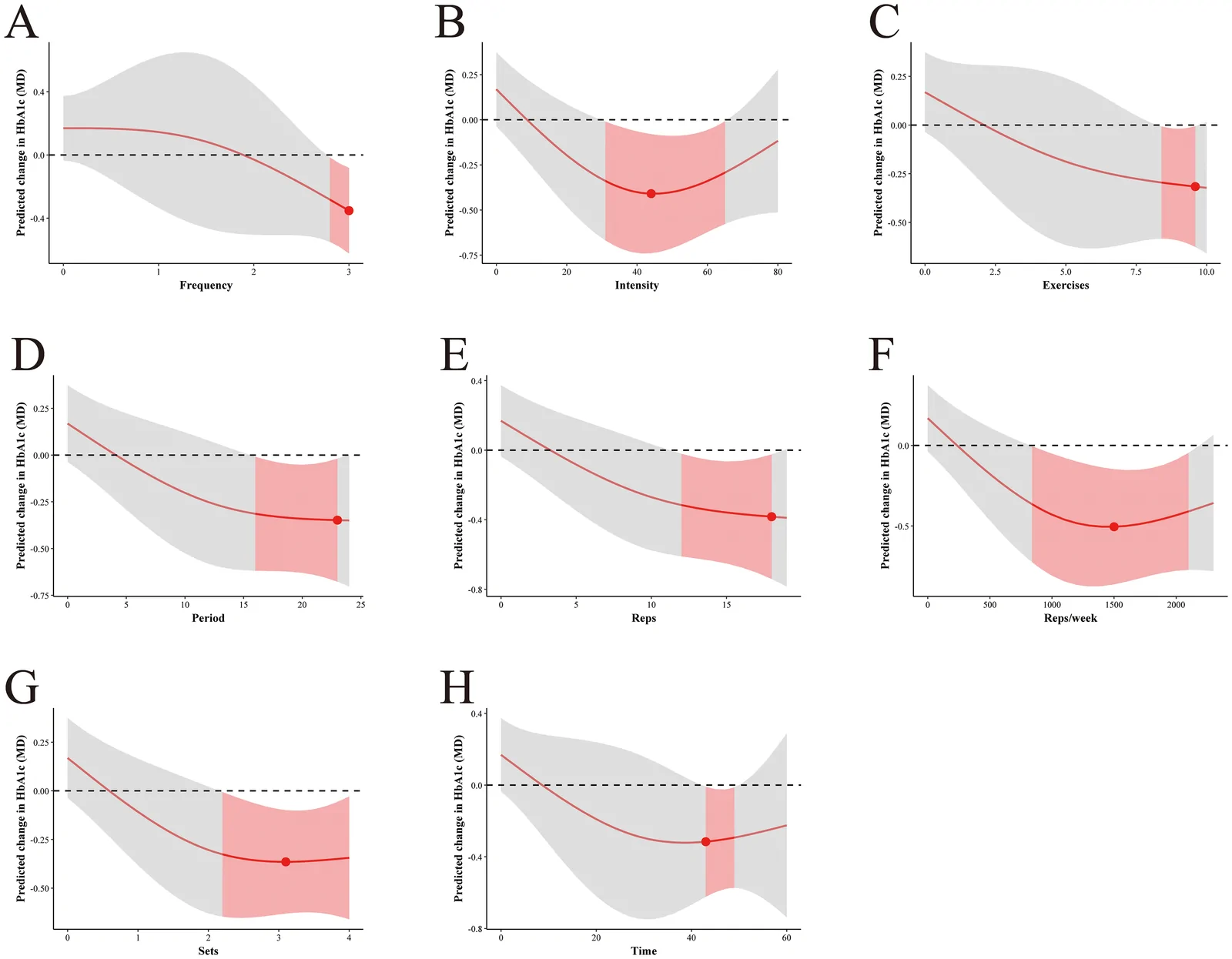
Dose-response relationship fitted using restricted cubic splines. **(A)** Association between resistance training frequency and HbA1c in overweight or obese patients with T2DM **(B)** Association between resistance training intensity and HbA1c in overweight or obese patients with T2DM **(C)** Association between the number of resistance training exercises and HbA1c in overweight or obese patients with T2DM **(D)** Association between resistance training peridization and HbA1c in overweight or obese patients with T2DM **(E)** Association between resistance training repetition count and HbA1c in overweight or obese patients with T2DM **(F)** Association between weekly resistance training volume and HbA1c in overweight or obese patients with T2DM **(G)** Association between the number of resistance training sets and HbA1c in overweight or obese patients with T2DM **(H)** Association between the duration of a single resistance training session and HbA1c in overweight or obese patients with T2DM. The red line represents the estimated hazard ratio, and the red shaded area corresponds to the credible interval, and the red dots indicate the optimal effective dose.

We employed the restricted cubic spline (RCS) function shown in **Figure 2A**, to investigate potential nonlinearities in the relationship between resistance training frequency and HbA1c in overweight or obese patients with T2DM. We found that resistance training frequency was associated with improvements in HbA1c among overweight or obese adults with T2DM. The observed dose-response relationship exhibited a nonlinear pattern: as the training frequency increased from low levels to approximately 3 times/week, the predicted HbA1c levels continued to decline (i.e., the improvement effect increased); once the frequency exceeded three times per week, the downward trend in HbA1c levels leveled off, and no further significant improvement was observed. Predicted maximal significant response was observed at 3 times/week (MD=-0.35, 95% CrI [-0.62, -0.08]). Furthermore, A training frequency of at least 3 times/week is the effective dose range for resistance training to significantly reduce glycated hemoglobin (HbA1c) in overweight or obese patients with type 2 diabetes.

#### 3.2.2 The effect of resistance training intensity on HbA1c levels in overweight/obese patients with T2DM

**Figure 2B** illustrates the dose-response relationships of resistance training intensity and HbA1c in overweight or obese patients with T2DM based on RCSs. The RCS analysis revealed an U-shaped curve for the relationship between resistance training intensity and HbA1c in overweight or obese patients with T2DM. Predicted maximal significant response was observed at 44% 1RM (MD=-0.41, 95% CrI [-0.74, -0.08]). Our results showed that 30%-62% 1RM was an effective intensity dose range for resistance training to significantly decrease HbA1c in overweight or obese patients with T2DM.

#### 3.2.3 The effect of resistance training exercises on HbA1c levels in overweight/obese patients with T2DM

Using RCS analysis, we investigated the dose-response relationship between the number of resistance training exercises and HbA1c levels in overweight or obese patients with T2DM (**Figure 2C**). Unlike the inverted U-shaped or U-shaped nonlinear relationships observed for resistance training frequency (**Figure 2A**) and intensity (**Figure 2B**), the number of exercises exhibited an approximately monotonic negative correlation with HbA1c. The analysis revealed that as the number of exercises included in each training session increased, predicted HbA1c levels showed a continuous downward trend, indicating a corresponding enhancement in glycemic control. The maximum predicted reduction in HbA1c was observed when each training session included 10 exercises (MD = −0.32, 95% CrI [−0.63, −0.01]). Furthermore, performing at least 8 exercises per session constitutes the effective dose range for resistance training to significantly reduce HbA1c levels in overweight or obese patients with T2DM.

#### 3.2.4 The effect of resistance training periodization on HbA1c levels in overweight/obese patients with T2DM

We used the RCS method to analyze the dose-response relationship between the total duration of resistance training and HbA1c levels in overweight or obese patients with T2DM (**Figure 2D**). The analysis revealed a significant nonlinear association between the two variables. Prior to reaching the point of maximum effect, longer training durations were associated with greater reductions in HbA1c, and there was an optimal total duration that yielded the greatest benefit. The predicted maximum reduction in HbA1c occurred at 23 weeks (MD = −0.35, 95% CrI [−0.68, −0.02]). Furthermore, resistance training programs lasting 17 to 23 weeks represent an effective dose range capable of significantly improving HbA1c levels in overweight or obese patients with T2DM.

#### 3.2.5 The effect of resistance training reps on HbA1c levels in overweight/obese patients with T2DM

To analyze the dose-response relationships between resistance training reps and HbA1c in overweight or obese patients with T2DM, a RCS model was used (**Figure 2E**). This dose-response curve resembles the patterns observed in **Figure 2C** and D, and likely exhibits an approximately linear, monotonically decreasing relationship. This implies that as the number of repetitions per training session increases, predicted HbA1c levels show a consistent downward trend. This suggests that, within a certain range, a higher training volume leads to greater improvements in glycemic control. Predicted maximal significant response was observed at 18 reps (MD=-0.38; 95% CrI [-0.74, -0.02]). Resistance training reps presented 13–18 reps was the effective dose range that achieve to improve HbA1c in overweight or obese patients with T2DM.

#### 3.2.6 The effect of resistance training volume on HbA1c levels in overweight/obese patients with T2DM

We used a restricted cubic spline (RCS) model to analyze the dose-response relationship between the total weekly volume of resistance training (measured as total weekly repetitions) and HbA1c levels in overweight or obese patients with T2DM (**Figure 2F**). The RCS analysis indicated a significant nonlinear association between the two variables, exhibiting a “diminishing returns” pattern (i.e., an “L-shaped” curve). Specifically, as the total weekly training volume increased, the predicted reduction in HbA1c gradually increased, reaching a maximum effect at approximately 1,500 reps/week (MD = −0.51, 95% CrI [−0.86, −0.15]). Thereafter, as training volume continued to increase, the growth in benefits tended to level off. Resistance training involving approximately 1000 to 2100 repetitions per week represents an effective dose range capable of significantly improving HbA1c levels in overweight or obese patients with T2DM.

#### 3.2.7 The effect of resistance training sets on HbA1c levels in overweight/obese patients with T2DM

We used an RCS model to analyze the dose-response relationship between the number of resistance training sessions and HbA1c levels in overweight or obese patients with T2DM (**Figure 2G**). The analysis indicated a significant nonlinear association between the two. Prior to reaching the optimal number of sessions, an increase in the number of training sessions was associated with a greater reduction in HbA1c. The predicted maximum reduction in HbA1c occurred when 3 sets (MD=−0.36, 95% CrI [−0.63, −0.10]). Based on the dose-response curve, performing 2–4 sets per training session represents an effective dose range capable of significantly improving HbA1c levels in overweight or obese patients with T2DM.

#### 3.2.8 The effect of resistance training time on HbA1c levels in overweight/obese patients with T2DM

We used an RCS model to analyze the dose-response relationship between the duration of resistance training and HbA1c levels in overweight or obese patients with T2DM (**Figure 2H**). The results presented in this figure (**Figure 2H**) indicate that, in overweight or obese patients with T2DM, there is a significant dose-response relationship between the duration of a single resistance training session and improvements in HbA1c, and this relationship exhibits a typical “U-shaped” curve. This implies that there is an optimal duration range, and training sessions that are either too long or too short may reduce the benefits for glycemic control. The predicted maximum reduction in HbA1c occurred was 45min (MD=−0.32, 95% CrI [−0.62, −0.01]). These findings underscore that, to optimize metabolic benefits in patients with T2DM, exercise prescriptions should precisely control the duration of each resistance training session to avoid sessions that are either too short or too long.

## 4 Discussion

To our knowledge, this is the first comprehensive dose-response meta-analysis conducted in overweight or obese patients with T2DM to investigate the relationship between resistance training dose (frequency, intensity, periodization, time, number of exercises, repetitions, sets, and training volume) and HbA1c. The analysis ultimately included 10 eligible randomized controlled trials involving 633 participants. This study yielded several key findings that are of critical importance to clinicians. First, The analysis revealed that all resistance training parameters examined (frequency, intensity, duration, training volume, etc.) were significantly associated with improvements in HbA1c, confirming the effectiveness of resistance training as a non-pharmacological intervention for glycemic control in patients with T2DM. More importantly, while the number of exercises and repetitions showed a nearly monotonic negative correlation, the dose-response curves for most parameters exhibited nonlinear characteristics (such as inverted U-shaped, U-shaped, and L-shaped curves). Second, this dose-response meta-analysis showed that the effective dose range for frequency of resistance training was 3 times/week, resistance training intensity was 30-62% 1RM, 17–23 weeks, 10 exercises per set, resistance training repetitions was 13–18 reps, 3 sets, resistance training volume was 1000–2100 reps per week. Third, the analysis suggested a potential optimum around of resistance training to improve HbA1c in overweight or obese patients with T2DM is 3 times per week, 44% 1RM, 23 weeks, 10 exercises, 18 reps, 3 sets, 1500 reps/week. In summary, our findings provide dose–response estimates derived from Bayesian MBNMA, rather than a formal grading of evidence, and should be interpreted within the context of the modeling assumptions and the available evidence base.

Consistent with previous studies, our analysis confirms that resistance training can reduce HbA1c levels in overweight or obese patients with type 2 diabetes (Jansson et al., 2022). This is because the abnormal glucose metabolism in patients with T2DM primarily manifests as changes in peripheral glucose metabolism; since skeletal muscle is the main peripheral site where insulin stimulates glucose uptake, skeletal muscle mass becomes a key influencing factor (Codella et al., 2018). The American Diabetes Association recommends that people with T2DM engage in at least 150 minutes of moderate- to vigorous-intensity aerobic exercise per week, as well as resistance training on 2–3 non-consecutive days. This is largely consistent with our findings. Our research indicates that for overweight or obese adults with T2DM, the optimal RT frequency for improving HbA1c levels is 3 times a week for 45 minutes each session. When training frequency exceeds 3 times per week or a single session exceeds 45 minutes, improvements in HbA1c no longer increase significantly and may even decline (Colberg et al., 2010; Su et al., 2023). This suggests that excessively high frequency or excessively long training sessions may increase fatigue, the risk of injury, or affect adherence (Colberg et al., 2010), thereby undermining metabolic benefits. Therefore, a recommended regimen of 3 sessions per week, each lasting 45 minutes, may represent the optimal balance between efficacy and risk.

Resistance training intensity exhibits a unique “U-shaped curve” relationship with HbA1c improvement, with the optimal intensity being 44% of 1RM and an effective range of 30%–62% of 1RM. It should be noted that the number of repetitions is typically used as a measure of training intensity, while the percentage of one’s 1RM determines the actual number of repetitions performed in each set before reaching muscle failure (Silva et al., 2014). As the number of repetitions per training session increased, the predicted HbA1c levels showed a consistent downward trend. Our study observed the greatest significant effect on glycemic control at 18 repetitions (<65% 1RM). This finding challenges the traditional view that “higher intensity is always better” (Vincent et al., 2002; Beneka et al., 2005). For overweight or obese patients with T2DM, moderate-intensity resistance training may strike the optimal balance between muscle metabolic activation, improved insulin sensitivity, and cardiovascular safety (Smith et al., 2023; Al-Mhanna et al., 2025). Excessively high intensity (>80% 1RM) may lead to excessive stress and safety concerns, while excessively low intensity may result in insufficient stimulation.

In our study, both the total duration of the intervention (23 weeks) and the training volume, as measured by 1,500 reps per week, exhibited a “diminishing returns”(L-shaped) pattern. During the initial phase of the exercise intervention (typically weeks 0-12), the body enters a period of rapid adaptation. During this phase, regular exercise significantly improves skeletal muscle glucose uptake efficiency and systemic insulin sensitivity, as evidenced by the most pronounced improvements in glycemic control markers (such as HbA1c). As the intervention continues to approximately 12–23 weeks, the body enters the pre-plateau phase. During this period, adaptive mechanisms shift from functional regulation to deeper structural optimization, such as increased muscle mitochondrial density and refinement of the capillary network. Although the absolute magnitude of improvement in blood glucose indicators remains significant, the rate of improvement has slowed markedly, indicating that the marginal benefits of the foundational adaptation phase are beginning to diminish. Once the intervention duration exceeds a certain threshold (e.g., 20–24 weeks), the body typically enters a phase of diminishing returns. At this point, the body has fully adapted to the established exercise regimen and established a new, optimized glucose metabolic set point. During this phase, simply increasing exercise duration or maintaining the original training regimen often fails to yield additional linear benefits. To break through this plateau, structural adjustments to the exercise prescription are necessary, such as significantly increasing exercise intensity or introducing new training modalities, to provide new physiological stressors and reactivate adaptive signaling pathways.

Within the parameters of this study, resistance training volume based on repetitions (defined as: training frequency × number of exercises × number of sets × number of repetitions) showed a significant dose-response relationship with improvements in HbA1c. This metric serves as an effective proxy for total training stimulus exposure in protocols with relatively fixed workload. Specifically, increasing the number of exercises (10) and the number of repetitions (18 reps) consistently yielded positive metabolic benefits within the observed range; however, increasing the number of sets (3 sets) showed a trend of diminishing returns, consistent with the principle of training moderation. A comprehensive evaluation indicates that when the total weekly training volume reaches approximately 1,500 repetitions, the effect on glycemic control plateaus, and the marginal benefits of further increasing training volume decrease significantly. This suggests that the body may have fully adapted to the current training regimen, reaching a new metabolic steady state, consistent with the law of diminishing returns observed in long-term exercise interventions. This finding suggests that when developing exercise prescriptions, one should seek the optimal range of training volume while ensuring proper form and intensity, rather than blindly pursuing an infinite accumulation of volume.

This study offers significant value and innovation, primarily reflected in the following aspects: First, multidimensional dose integration: Unlike previous studies that mostly focused on a single parameter (such as frequency or intensity alone), this study is the first to systematically integrate eight core dose parameters of resistance training through a network meta-analysis. It evaluated their combined dose-response relationships, providing a more comprehensive exercise prescription framework that better aligns with real-world training scenarios. Second, focus on a specific population: This study specifically targeted “overweight or obese patients with type 2 diabetes (T2DM),” a group characterized by a heavier metabolic burden and potentially lower exercise tolerance. The findings—such as the optimal intensity of 44% of 1RM at moderate intensity—are likely better aligned with the physiological characteristics and safety requirements of this population, demonstrating stronger clinical relevance. Furthermore, the methodology is rigorous: the use of Model-Based Network Meta-Analysis (MBNMA) within a Bayesian framework and Restricted Cubic Splines (RCS) for nonlinear dose-response modeling enables more precise capture of the complex relationships between parameters and outcomes, providing parameter rankings and estimates of optimal dose points with probabilistic significance.

This study provides a clear, evidence-based “prescription” for clinicians and exercise therapists to develop resistance training programs for overweight/obese patients with type 2 diabetes. The recommended protocol—”23 weeks, 3 sessions per week, 45 minutes per session, 10 exercises, 3 sets of 18 repetitions, at 44% of 1RM”—can serve as a standard intervention template. Additionally, the provided “effective dose range” allows for personalized adjustments based on individual patient factors (such as baseline fitness, comorbidities, and scheduling), thereby enhancing the feasibility and adherence of the prescription.

This study identifies several directions for future research: (1) Mechanism exploration: Why is moderate intensity (44% 1RM) superior to high intensity? The underlying molecular mechanisms (such as myokine secretion and the efficiency of insulin signaling pathway activation) require further investigation. (2) Optimization of combinations: This study analyzed the independent effects of each parameter; future research should employ factorial designs to explore interactions between different parameters (such as intensity and number of sets) to identify more refined combination strategies. (3) Long-term effects and adherence: While 23 weeks was determined to be the optimal duration, the effects of longer durations (such as 1 year or more), the maintenance of these effects, and how to sustain high adherence in real-life settings are practical issues that need to be addressed. (4) Population Heterogeneity: Future studies could further analyze subgroups within the population defined in this study—based on age, gender, duration of diabetes, and degree of obesity—to provide more precise subgroup-specific treatment recommendations. (5) A further limitation is the lack of subgroup analyses based on BMI cutoff categories (e.g., international vs. Asian definitions of overweight and obesity). Although BMI criteria varied across included studies, the limited number of trials and considerable clinical and methodological heterogeneity precluded meaningful subgroup comparisons. Future meta-analyses with a larger evidence base are encouraged to explore whether treatment effects differ according to BMI classification systems. A further limitation is that we did not systematically search gray literature sources, such as clinical trial registries (e.g., ClinicalTrials.gov, WHO International Clinical Trials Registry Platform), dissertation databases (e.g., ProQuest Dissertations & Theses Global), or conference abstracts. Although our search of major electronic databases captured the majority of peer-reviewed randomized controlled trials, the exclusion of gray literature may lead to the omission of unpublished or negative studies. This could introduce publication bias and potentially result in an overestimation of the true treatment effects. Future meta-analyses with a larger evidence base are encouraged to incorporate systematic gray literature searches to mitigate this bias and provide more robust estimates of the dose–response relationship between resistance training and HbA1c in overweight and obese patients with type 2 diabetes. Nevertheless, the consistency of our findings with previously published meta-analyses suggests that the impact of this limitation on our primary conclusions is likely modest.

## 5 Conclusions

This systematic review and meta-analysis revealed a dose-response relationship between various variables of resistance training and HbA1c levels in overweight or obese patients with T2DM, confirming our preliminary hypothesis that each training variable has an optimal range that maximizes improvements in HbA1c levels. Our findings support the conclusion that resistance training is effective in improving HbA1c levels in overweight or obese patients with T2DM. The recommended 23-week training program (MD = −0.35, 95% CrI [−0.68, −0.02]) includes training 3 times per week (MD=-0.35, 95% CrI [-0.62, -0.08]), 45min per session (MD = −0.32, 95% CrI [−0.62, −0.01]) at an intensity of 44% of 1RM (MD=-0.41, 95% CrI [-0.74, -0.08]), with 10 exercises per set MD = −0.32, 95% CrI [−0.63, −0.01]), for a total of 3 sets (MD=−0.36, 95% CrI [−0.63, −0.10]), 18 reps per exercise (MD=-0.38; 95% CrI [-0.74, -0.02]), and a total of 1,500 reps per week (MD = −0.51, 95% CrI [−0.86, −0.15]). Furthermore, errors in data recording and the process of fitting models to experimental data may introduce uncertainty into the estimates. Therefore, this study aims to establish quantitative evidence regarding the relationship between resistance training dose and HbA1c improvement to guide overweight or obese patients with T2DM in scientifically conducting resistance training to control their HbA1c levels.

## Data Availability

The datasets presented in this study can be found in online repositories. The names of the repository/repositories and accession number(s) can be found in the article/**Supplementary Material**.
