# Supplementary material for: Optimal exercise dose for resistance training to improve HbA1c levels in overweight and obese patients with type 2 diabetes: a systematic review of randomized controlled trials and a Bayesian network meta-analysis
Source: Front Physiol. 2026 Aug 12;17:1867073. doi: 10.3389/fphys.2026.1867073 (PMC13506765; doi:10.3389/fphys.2026.1867073)
Supplement: Supplementary file 1 [file Table1.docx]

**Optimal Exercise Dose for Improving HbA1c in Overweight and Obese Patients with T2DM : A systematic review and Bayesian model-based network meta-analysis of RCTs**

**Table of Contents**

[Supplementary file 1: Search strategy	2](#_Toc2625)

[Supplementary file 2: Characteristics of included studies	7](#_Toc11570)

[Supplementary file 3: Key assumptions of Network Meta-Analysis	10](#_Toc22926)

[*Connectivity*	10](#_Toc26398)

[Supplementary Figure 1. Treatment-level network.	10](#_Toc20466)

[*Consistency*	11](#_Toc17559)

[Supplementary Table 1. Consistent and UME models fit comparison	11](#_Toc28216)

[*Transitivity*	13](#_Toc23514)

[Supplementary Table 2. Node-splitting analysis of inconsistency	13](#_Toc4054)

[Supplementary Figures 2. Node-splitting analysis (density plot).	18](#_Toc2024)

[Supplementary file 4: Non-linear functions and models fit comparison	19](#_Toc909)

[Supplementary Figure 3. “Split” NMA of different Exercise dose.	19](#_Toc17727)

[Supplementary Table 3. Models fit comparison	20](#_Toc438)

[Supplementary Figure 4. The deviance plot for treatment effects.	27](#_Toc10586)

[Supplementary file 5: Characteristics of included studies	28](#_Toc13889)

[List of included studies	30](#_Toc29511)

[References	31](#_Toc887)

# Supplementary file 1: Search strategy

| PubMed  (2026-01-19) | 1. ((((((((((((((((((((((((Physical activity[MeSH Terms]) OR (Physical activity[Title/Abstract])) OR (Exercise[Title/Abstract])) OR (Exercise[MeSH Terms])) OR (sport[MeSH Terms])) OR (sport[Title/Abstract])) OR (Endurance activity[Title/Abstract])) OR (Endurance activities[Title/Abstract])) OR (Aerobic activities[Title/Abstract])) OR (Aerobic activity[Title/Abstract])) OR (resistance training;strength training[Title/Abstract])) OR (Muscle-strength[Title/Abstract])) OR (weight-lifting program[Title/Abstract])) OR (high-intensity interval training[Title/Abstract])) OR (HIIT[Title/Abstract])) OR (yoga[Title/Abstract])) OR (walking[Title/Abstract])) OR (running[Title/Abstract])) OR (swimming[Title/Abstract])) OR (cycling[Title/Abstract])) OR (multicomponent[Title/Abstract])) OR (mixed aerobic exercise[Title/Abstract])) OR (aichi[Title/Abstract])) OR (physical fitness[Title/Abstract])) OR (physical fitness[Title/Abstract]) 2. ((((obesity[MeSH Terms]) OR (obesity[Title/Abstract])) OR (fat[Title/Abstract])) OR (Overweight[Title/Abstract])) OR (Obese[Title/Abstract]) 3. (((((((Diabetes Mellitus[MeSH Terms]) OR (Diabetes Mellitus[Title/Abstract])) OR (Type 2;Diabetes Mellitus[Title/Abstract])) OR (Type 2;Diabetes Mellitus[MeSH Terms])) OR (Diabetes Mellitus, type II[MeSH Terms])) OR (Diabetes Mellitus, type II[Title/Abstract])) OR (Type 2 diabetes[Title/Abstract])) OR (type II diabetes[Title/Abstract]) 4. (((((obesity[MeSH Terms]) OR (obesity[Title/Abstract])) OR (fat[Title/Abstract])) OR (Overweight[Title/Abstract])) OR (Obese[Title/Abstract])) AND ((((((((Diabetes Mellitus[MeSH Terms]) OR (Diabetes Mellitus[Title/Abstract])) OR (Type 2;Diabetes Mellitus[Title/Abstract])) OR (Type 2;Diabetes Mellitus[MeSH Terms])) OR (Diabetes Mellitus, type II[MeSH Terms])) OR (Diabetes Mellitus, type II[Title/Abstract])) OR (Type 2 diabetes[Title/Abstract])) OR (type II diabetes[Title/Abstract])) 5. ((((((((patient[MeSH Terms]) OR (patients[MeSH Terms])) OR (adult[MeSH Terms])) OR (adult[Title/Abstract])) OR (Aged[Title/Abstract])) OR (older[Title/Abstract])) OR (young adult[Title/Abstract])) OR (person[Title/Abstract])) OR (Middle aged[Title/Abstract]) 6. (((((((Teenager[MeSH Terms]) OR (Teenagers[Title/Abstract])) OR (Adolescent[MeSH Terms])) OR (Adolescent[Title/Abstract])) OR (youth[Title/Abstract])) OR (youth[MeSH Terms])) OR (Adolescents[Title/Abstract])) OR (Youths[Title/Abstract]) 7. ((((((mouse[Title/Abstract]) OR (animal[MeSH Terms])) OR (mice[Title/Abstract])) OR (Rat[Title/Abstract])) OR (mices[Title/Abstract])) OR (rats[Title/Abstract])) OR (mouses[Title/Abstract]) 8. ((((((((((patient[MeSH Terms]) OR (patients[MeSH Terms])) OR (adult[MeSH Terms])) OR (adult[Title/Abstract])) OR (Aged[Title/Abstract])) OR (older[Title/Abstract])) OR (young adult[Title/Abstract])) OR (person[Title/Abstract])) OR (Middle aged[Title/Abstract])) NOT ((((((((Teenager[MeSH Terms]) OR (Teenagers[Title/Abstract])) OR (Adolescent[MeSH Terms])) OR (Adolescent[Title/Abstract])) OR (youth[Title/Abstract])) OR (youth[MeSH Terms])) OR (Adolescents[Title/Abstract])) OR (Youths[Title/Abstract]))) NOT (((((((mouse[Title/Abstract]) OR (animal[MeSH Terms])) OR (mice[Title/Abstract])) OR (Rat[Title/Abstract])) OR (mices[Title/Abstract])) OR (rats[Title/Abstract])) OR (mouses[Title/Abstract])) 9. (randomized controlled trial[pt] OR controlled clinical trial[pt] OR randomized[tiab] OR placebo[tiab] OR drug therapy[sh] OR randomly[tiab] OR trial[tiab] OR groups[tiab]) NOT (animals[mh] NOT humans[mh]) 10. ((((((((obesity[MeSH Terms]) OR (obesity[Title/Abstract])) OR (fat[Title/Abstract])) OR (Overweight[Title/Abstract])) OR (Obese[Title/Abstract])) AND ((((((((Diabetes Mellitus[MeSH Terms]) OR (Diabetes Mellitus[Title/Abstract])) OR (Type 2;Diabetes Mellitus[Title/Abstract])) OR (Type 2;Diabetes Mellitus[MeSH Terms])) OR (Diabetes Mellitus, type II[MeSH Terms])) OR (Diabetes Mellitus, type II[Title/Abstract])) OR (Type 2 diabetes[Title/Abstract])) OR (type II diabetes[Title/Abstract]))) AND (((((((((((((((((((((((((Physical activity[MeSH Terms]) OR (Physical activity[Title/Abstract])) OR (Exercise[Title/Abstract])) OR (Exercise[MeSH Terms])) OR (sport[MeSH Terms])) OR (sport[Title/Abstract])) OR (Endurance activity[Title/Abstract])) OR (Endurance activities[Title/Abstract])) OR (Aerobic activities[Title/Abstract])) OR (Aerobic activity[Title/Abstract])) OR (resistance training;strength training[Title/Abstract])) OR (Muscle-strength[Title/Abstract])) OR (weight-lifting program[Title/Abstract])) OR (high-intensity interval training[Title/Abstract])) OR (HIIT[Title/Abstract])) OR (yoga[Title/Abstract])) OR (walking[Title/Abstract])) OR (running[Title/Abstract])) OR (swimming[Title/Abstract])) OR (cycling[Title/Abstract])) OR (multicomponent[Title/Abstract])) OR (mixed aerobic exercise[Title/Abstract])) OR (aichi[Title/Abstract])) OR (physical fitness[Title/Abstract])) OR (physical fitness[Title/Abstract]))) AND (((((((((((patient[MeSH Terms]) OR (patients[MeSH Terms])) OR (adult[MeSH Terms])) OR (adult[Title/Abstract])) OR (Aged[Title/Abstract])) OR (older[Title/Abstract])) OR (young adult[Title/Abstract])) OR (person[Title/Abstract])) OR (Middle aged[Title/Abstract])) NOT ((((((((Teenager[MeSH Terms]) OR (Teenagers[Title/Abstract])) OR (Adolescent[MeSH Terms])) OR (Adolescent[Title/Abstract])) OR (youth[Title/Abstract])) OR (youth[MeSH Terms])) OR (Adolescents[Title/Abstract])) OR (Youths[Title/Abstract]))) NOT (((((((mouse[Title/Abstract]) OR (animal[MeSH Terms])) OR (mice[Title/Abstract])) OR (Rat[Title/Abstract])) OR (mices[Title/Abstract])) OR (rats[Title/Abstract])) OR (mouses[Title/Abstract])))) AND ((randomized controlled trial[pt] OR controlled clinical trial[pt] OR randomized[tiab] OR placebo[tiab] OR drug therapy[sh] OR randomly[tiab] OR trial[tiab] OR groups[tiab]) NOT (animals[mh] NOT humans[mh])) |
| --- | --- |
| Embase (2026-01-19) | 1. 'obesity'/exp OR 'obesity' OR 'obesity':ti,ab,kw OR 'fat':ti,ab,kw OR obese:ti,ab,kw 2. 'diabetes mellitus'/exp OR 'diabetes mellitus' OR 'non insulin dependent diabetes 3. #1 AND #2 4. 'physical activity'/exp OR 'physical activity' OR 'exercise':ti,ab,kw OR 'sport':ti,ab,kw OR 'endurance training':ti,ab,kw OR 'aerobic exercise':ti,ab,kw OR 'resistance training':ti,ab,kw OR 'muscle strength':ti,ab,kw OR 'weight training':ti,ab,kw OR 'high intensity interval training':ti,ab,kw OR 'yoga':ti,ab,kw OR 'walking':ti,ab,kw OR running:ti,ab,kw OR swimming:ti,ab,kw OR 'cycling':ti,ab,kw OR 'multicomponent training':ti,ab,kw OR 'fitness':ti,ab,kw OR 'dancing':ti,ab,kw 5. ('patient'/exp OR 'patient' OR 'adult':ti,ab,kw OR 'aged':ti,ab,kw OR 'older adults':ti,ab,kw OR 'young adult':ti,ab,kw OR 'middle aged':ti,ab,kw) NOT 'adolescent':ti,ab,kw NOT 'juvenile':ti,ab,kw NOT 'mouse':ti,ab,kw NOT animal:ti,ab,kw NOT 'rat':ti,ab,kw 6. #3 AND #4 AND #5 7. ('crossover procedure':de OR 'double-blind procedure':de OR 'randomized controlled trial':de) AND or  AND 'single-blind procedure':de OR (random*:de,ab,ti AND or:de,ab,ti AND factorial*:de,ab,ti) OR crossover*:de,ab,ti OR ((cross NEXT/1 over*):de,ab,ti) OR placebo*:de,ab,ti OR ((doubl* NEAR/1 blind*):de,ab,ti) OR ((singl* NEAR/1 blind*):de,ab,ti) OR assign*:de,ab,ti OR allocat*:de,ab,ti OR volunteer*:de,ab,ti 8. #6 AND #7 |
| Cochrane (2026-01-19) | 1. (“Diabetes Mellitus”):ti,ab,kw OR ("Diabetes MellitusType II"):ti,ab,kw OR ("Diabetes Mellitus type 2"):ti,ab,kw OR ("type 2 diabetes mellitus"):ti,ab,kw OR ("Type II diabetes mellitus"):ti,ab,kw (Word variations have been searched) 89221 2. (“OBESITY”):ti,ab,kw OR ("obese"):ti,ab,kw OR ("fat"):ti,ab,kw OR ("overweight"):ti,ab,kw (Word variations have been searched) 98610 3. #3 #1 and #2 14799 4. #4 (“Physical activity”):ti,ab,kw OR ("Physical activities"):ti,ab,kw OR ("sport"):ti,ab,kw OR ("sports"):ti,ab,kw OR (“exercise”):ti,ab,kw (Word variations have been searched) 194062 5. (“Endurance activity”):ti,ab,kw OR ("Physical activities"):ti,ab,kw OR ("strength training"):ti,ab,kw OR ("Aerobic activity"):ti,ab,kw OR (“resistance training”):ti,ab,kw (Word variations have been searched) 68267 6. (“Muscle-strength”):ti,ab,kw OR ("weight-lifting program"):ti,ab,kw OR ("high-intensity interval training"):ti,ab,kw OR ("HIIT"):ti,ab,kw OR (“yoga”):ti,ab,kw (Word variations have been searched) 11078 7. (“walking”):ti,ab,kw OR ("swimming"):ti,ab,kw OR ("running"):ti,ab,kw OR ("cycling"):ti,ab,kw OR (“taichi”):ti,ab,kw (Word variations have been searched) 155520 8. (“physical fitness”):ti,ab,kw OR ("dancing"):ti,ab,kw OR ("mixed aerobic exercise"):ti,ab,kw OR ("multicomponent"):ti,ab,kw (Word variations have been searched) 14501 9. #4 or #5 or #6 or #7 or #8 314495 10. (“patients”):ti,ab,kw OR ("adult"):ti,ab,kw OR ("Aged"):ti,ab,kw OR ("older"):ti,ab,kw OR ("Middle aged"):ti,ab,kw (Word variations have been searched) 1813736 11. (“Teenagers”):ti,ab,kw OR ("Adolescent"):ti,ab,kw OR ("youth"):ti,ab,kw OR ("Adolescents"):ti,ab,kw OR ("Youths"):ti,ab,kw (Word variations have been searched) 183627 12. #10 not #11 1648422 13. #3 and #9 and #12 3575 |

# Supplementary file 2: Characteristics of included studies

| Study ID | Age  (Mean±SD) | Sample Size  (M/F) | HbA1c (%) | BMI | Diagnosis of Type 2 diabetes | Intervention detail |
| --- | --- | --- | --- | --- | --- | --- |
| Soumitra Mondal et al. (2021) | 40-64 | CON: 6(-/-) RT: 6(-/-) | CON: 8.8±2.1  RT: 8.7±1.5 | BMI＜35kg/m^2^ | HbA1c levels between 6.5% and 11.0% | RT: 16weeks, 40 min, 3 times/week, 6 exercises, 70%-80% 1RM, 8-11 reps/set, 3-4 sets. |
| Xiaojun Ma et al. (2024) | CON: 56.29±5.92  RT: 57.56±4.85 | CON: 32(16/16) RT: 31(11/20) | CON: 8.23±1.02  RT: 8.01±0.83 | CON: 26.92±1.74  RT: 26.33±1.76 | HbA1c levels between 6.5% and 11.0% | RT: 12weeks, 40 min, 3 times/week, 9 exercises, 20%-40% 1RM, 15-30 reps/set, 4 sets. |
| Xiaojun Ma et al. (2024) | CO: 65.55±4.41  RT: 66.65±4.94 | CON: 33(14/19) RT: 31(13/18) | CON: 7.98±0.96  RT:7.80±0.93 | CON: 26.56±1.58  RT: 26.02±2.05 | HbA1c levels between 6.5% and 11.0% | RT: 12weeks, 40min, 3 times/week, 10 exercises, 40%-60% 1RM, 8-15 reps/set, 2-4 sets. |
| Xiaojun Ma et al. (2024) | CON: 65.55±4.41  RT: 66.41±4.97 | CON: 33(14/19) RT: 34 (12/22) | CON: 7.98±0.96  RT: 7.75±0.97 | CON: 26.56±1.58  RT: 26.31±1.88 | HbA1c levels between 6.5% and 11.0% | RT: 24weeks, 40min, 3 times/week, 10 exercises, 20%-30% 1RM, 15-30 reps/set, 2-4 sets. |
| Nikolaos P. E. Kadoglou et al. (2020) | CON：64.6±4.3  RT：61.5±5.4 | CON: 24(5/19) RT: 23(7/16) | CON：7.5±0.5  RT：7.4±0.4 | CON：31.58±5.71  RT：32.74±4.05 | HbA1c levels ＞6.5 % | RT: 12weeks, 45 min-60 min, 3 times/week, 8 exercises, 60%-80% 1RM, 6-8 reps/set, 2-3sets. |
| Jürgen Giessing et al. (2022) | CON：64±7  RT：61±5 | CON: 28(-/-) RT: 29(-/-) | CON：8.3±0.7  RT：8.4±0.9 | CON：31.7±5.3  RT：32.1±6.3 | HbA1c level ≥6.3%; FBG≥100mg/dl | RT: 24weeks, 45 min, 2 times/week, 10 exercises, 67%-80% 1RM, 8-12 reps/set, 1 set. |
| CARMEN CASTANEDA et al.(2002) | CON: 66±1  RT: 66±2 | CON: 31(12/19) RT: 31(10/21) | CON: 8.4±0.3  RT: 8.7±0.3 | CON: 31.2±1.0  RT: 30.9±1.1 | FBG≥7.0 mmol/l | RT: 16weeks, 45 min, 3 times/week, 6 exercises, 60%-80% 1RM, 8 reps/set, 3 sets. |
| J. C. Baldi et al.(2003) | CON: 50.1±1.3  RT: 46.5±2.1 | CON: 9(9/0) RT: 9(9/0) | CON: 8.5±0.8  RT: 8.9±1.2 | CON: 36.4±3.1  RT: 34.3±3.2 | NA | RT: 10weeks, 40 min, 3 times/week, 10 exercises, 70% 1RM, 12 reps/set, 2 sets. |
| Ronald J. Sigal et al.(2016) | CON：54.8±7.2  RT：54.7±7.5 | CON: 63(41/22) RT: 64(40/24) | CON:7.66±0.89  RT: 7.71±0.86 | CON: 35.0±9.5  RT: 34.1±9.6 | HbA1c levels between 6.6% and 9.9%. American Diabetes Association | RT: 22weeks, 45 min, 3 times/week, 7 exercises, 80% 1RM, 8 reps/set, 2-3 sets. |
| Vanessa Neves de Oliveira et al.(2011) | CON：53.42±9.82  RT：54.10±8.94 | CON: 12(4/8) RT: 10(4/6) | CON:7.04±0.68  RT: 8.51±2.46 | CON: 30.03±4.90  RT: 31.29±4.08 | NA | RT: 12weeks, 60min, 3 times/week, 7 exercises, 50% 1RM, 8-12 reps/set, 4 sets. |
| N.W.Cheung et al.(2009) | CON：65.55±4.41  RT：66.65±4.94 | CON: 63(41/22)  RT: 64(40/24) | CON:7.4±1.0  RT: 7.2±1.6 | CON: 37.7±9.2  RT: 39.7±9.0 | NA | RT: 16weeks, 40 min, 3 times/week, 7 exercises, 67% 1RM, 12 reps/set, 2 sets. |

*Note.* M/F: Male or Female; RT: Resistance training; CON: control group; BMI: Body Mass Index; NA: Not available..

# Supplementary file 3: Key assumptions of Network Meta-Analysis

First, we plotted the observed effects of different resistance training doses on HbA1c levels. Based on the observed shapes, a range of recommended non-linear functions (i.e., Emax, restricted cubic splines, quadratic, and non-monotonically up^[1]^ were used to model the data. Next, we derived and compared different fit indices^[2]^ (i.e., Deviance Information Criterion (DIC), between-study standard deviation, number of parameters in the model, residual values) as well as corresponding deviance plots^[2]^ across models (Supplementary File 4). Restricted cubic splines yielded the best fit in all cases and were therefore used to assess the non-linear dose-response associations (Supplementary File 4). According to the model with the best fit (Supplementary Table 3) and biological plausibility^[1]^, we placed three knots at the 10th, 50th and 90th percentile of the exercise dose^[3, 4]^. Departure from linearity was assessed using a Wald test^[3, 4]^. Beta coefficients from the restricted cubic splines were used to estimate the resistance training dose at which the predicted maximal significant effect on HbA1c levels was achieved. This information was used to rank the doses of resistance training based on their probability to elicit changes on blood glucose levels, from worst to best.

### **Supplementary Figure 1.** Schematic diagram of network meta-statistics analysis

There are three key assumptions to conduct a Network Meta-Analysis (NMA): (1) network connectivity, (2) consistency in the data, and (3) transitivity.

*Connectivity*

Connectivity is a key assumption in NMA which if deemed insufficient (i.e., due to lack of direct comparators) can lead to low statistical power and misleading results (ter Veer E, et al., 2019). In our study, we assessed connectivity of the network at both treatment and agent levels visually and found no evidence of unconnectedness on either network (Supplementary Figure 1).

### **Supplementary Figure 2.** Treatment-level network.

**Supplementary Figure 2**. Network plot of study limitations of the included studies. (A): Frequency; (B): Intensity; (C):Exercises; (D): Period; (E): Reps; (F): Reps/week; (G): Sets; (H) time

*Consistency*

We carried out consistency analysis in the data through the comparison of consistent (i.e., network effect sizes) and unrelated mean effects (UME) models (i.e., pairwise effect sizes) of the network. In practice, we checked whether deviance, the number of estimated parameters in the network, and the Deviance Informative Criterion (DIC) indicators were similar for both models which would indicate a good fit (Wheeler DC, et al., 2010). Comparison of these parameters indicated good consistency across models (Supplementary Table 1).

## **Supplementary Table 1.** Consistent and UME models fit comparison

**Table 1.1.** Consistent and UME models fit comparison (Frequency)

| **Model** | **pD** | **Residual deviance** | **Deviance** | **DIC** | **SD** |
| --- | --- | --- | --- | --- | --- |
| Consistent | 19.5 | 29.46 | -14.480 | 4.5 | 0.15 |
| UME | 19.0 | 29.69 | -14.257 | 4.3 | 0.14 |

**Table 1.2.** Consistent and UME models fit comparison (Intensity)

| **Model** | **pD** | **Residual deviance** | **Deviance** | **DIC** | **SD** |
| --- | --- | --- | --- | --- | --- |
| Consistent | 22.8 | 27.76 | -18.14 | 4.0 | 0.30 |
| UME | 21.6 | 27.53 | -18.36 | 2.7 | 0.34 |

**Table 1.3.** Consistent and UME models fit comparison (Exercises)

| **Model** | **pD** | **Residual deviance** | **Deviance** | **DIC** | **SD** |
| --- | --- | --- | --- | --- | --- |
| Consistent | 24 | 27.75 | -16.19 | 7.2 | 0.26 |
| UME | 21.8 | 27.69 | -16.26 | 5.0 | 0.28 |

**Table 1.4.** Consistent and UME models fit comparison (Period)

| **Model** | **pD** | **Residual deviance** | **Deviance** | **DIC** | **SD** |
| --- | --- | --- | --- | --- | --- |
| Consistent | 23.4 | 29.84 | -14.10 | 8.7 | 0.25 |
| UME | 21.7 | 27.53 | -16.41 | 4.7 | 0.28 |

**Table 1.5** Consistent and UME models fit comparison (Reps)

| **Model** | **pD** | **Residual deviance** | **Deviance** | **DIC** | **SD** |
| --- | --- | --- | --- | --- | --- |
| Consistent | 21.7 | 26.66 | -19.23 | 2.0 | 0.23 |
| UME | 21.2 | 26.42 | -19.50 | 1.2 | 0.27 |

**Table 1.6.** Consistent and UME models fit comparison (Reps/week)

| **Model** | **pD** | **Residual deviance** | **Deviance** | **DIC** | **SD** |
| --- | --- | --- | --- | --- | --- |
| Consistent | 20.6 | 23.19 | -24.33 | -4.3 | 0.19 |
| UME | 21.0 | 24.45 | -23.07 | -2.8 | 0.36 |

**Table 1.7** Consistent and UME models fit comparison (Sets)

| **Model** | **pD** | **Residual deviance** | **Deviance** | **DIC** | **SD** |
| --- | --- | --- | --- | --- | --- |
| Consistent | 18.2 | 25.63 | -21.89 | -4.2 | 0.13 |
| UME | 17.0 | 27.44 | -20.08 | -3.4 | 0.14 |

**Table 1.8** Consistent and UME models fit comparison (time)

| **Model** | **pD** | **Residual deviance** | **Deviance** | **DIC** | **SD** |
| --- | --- | --- | --- | --- | --- |
| Consistent | 22.2 | 25.47 | -20.11 | 1.4 | 0.23 |
| UME | 19.6 | 28.57 | -17.00 | 2.1 | 0.19 |

*Note.* pD: Number of estimated parameters; DIC: Deviance Informative Criterion; SD: Standard Deviation; UME: Unrelated Mean Effects. Scientific literature indicated that the main indicator to assess the model fit is the DIC. As lower DIC, better fit.

*Transitivity*

We assessed transitivity via MBNMA node-splitting approach. This method splits and compares contributions for a particular treatment contrast into direct and indirect evidence (van Valkenhoef et al., 2016). Similar effects denote good transitivity. Supplementary Figures 2 and Table 2 below present the results for transitivity in this meta-analysis.

## **Supplementary Table 2.** Node-splitting analysis of inconsistency

Table 2.1. Node-splitting analysis of inconsistency (Frequency)

| Comparison | p-value | Median | 2.50% | 97.50% |
| --- | --- | --- | --- | --- |
| RT_3 vs Placebo_0 | 0.309 |  |  |  |
| -> direct |  | -0.559 | -0.71 | -0.369 |
| -> indirect |  | -0.281 | -0.892 | 0.314 |
| -> MBNMA |  | -0.53 | -0.676 | -0.34 |
|  |  |  |  |  |
| RT_2 vs Placebo_0 | 0.329 |  |  |  |
| -> direct |  | -0.202 | -0.599 | 0.211 |
| -> indirect |  | -0.378 | -0.484 | -0.238 |
| -> MBNMA |  | -0.357 | -0.456 | -0.231 |

Table 2.2. Node-splitting analysis of inconsistency (Intensity)

| Comparison | p-value | Median | 2.50% | 97.50% |
| --- | --- | --- | --- | --- |
| RT_80 vs Placebo_0 | 0.247 |  |  |  |
| -> direct |  | -0.152 | -0.708 | 0.366 |
| -> indirect |  | -0.605 | -0.886 | -0.317 |
| -> MBNMA |  | -0.523 | -0.777 | -0.261 |
| RT_70 vs Placebo_0 | 0.871 |  |  |  |
| -> direct |  | -0.471 | -0.889 | -0.04 |
| -> indirect |  | -0.518 | -0.828 | -0.145 |
| -> MBNMA |  | -0.496 | -0.726 | -0.245 |
| RT_50 vs Placebo_0 | 0.523 |  |  |  |
| -> direct |  | -0.562 | -0.976 | 0.039 |
| -> indirect |  | -0.398 | -0.625 | -0.146 |
| -> MBNMA |  | -0.428 | -0.622 | -0.202 |
| RT_45 vs Placebo_0 | 0.538 |  |  |  |
| -> direct |  | -0.541 | -1.118 | 0.016 |
| -> indirect |  | -0.377 | -0.6 | -0.12 |
| -> MBNMA |  | -0.405 | -0.598 | -0.188 |
| RT_25 vs Placebo_0 | 0.301 |  |  |  |
| -> direct |  | -0.565 | -1.068 | -0.03 |
| -> indirect |  | -0.247 | -0.478 | -0.093 |
| -> MBNMA |  | -0.285 | -0.512 | -0.122 |

Table 2.3. Node-splitting analysis of inconsistency (Exercises)

| Comparison | p-value | Median | 2.50% | 97.50% |
| --- | --- | --- | --- | --- |
| RT_10 vs Placebo_0 | 0.786 |  |  |  |
| -> direct |  | -0.504 | -0.814 | -0.191 |
| -> indirect |  | -0.579 | -0.941 | -0.132 |
| -> MBNMA |  | -0.532 | -0.758 | -0.291 |
| RT_9 vs Placebo_0 | 0.627 |  |  |  |
| -> direct |  | -0.549 | -1.054 | -0.03 |
| -> indirect |  | -0.467 | -0.711 | -0.218 |
| -> MBNMA |  | -0.485 | -0.687 | -0.266 |
| RT_8 vs Placebo_0 | 0.402 |  |  |  |
| -> direct |  | -0.631 | -1.334 | 0.136 |
| -> indirect |  | -0.427 | -0.627 | -0.203 |
| -> MBNMA |  | -0.437 | -0.623 | -0.24 |
| RT_7 vs Placebo_0 | 0.197 |  |  |  |
| -> direct |  | -0.023 | -0.53 | 0.503 |
| -> indirect |  | -0.429 | -0.618 | -0.251 |
| -> MBNMA |  | -0.386 | -0.553 | -0.213 |
| RT_6 vs Placebo_0 | 0.135 |  |  |  |
| -> direct |  | -1.114 | -3.117 | 0.842 |
| -> indirect |  | -0.332 | -0.487 | -0.172 |
| -> MBNMA |  | -0.334 | -0.483 | -0.184 |
| RT_5 vs Placebo_0 | 0.099 |  |  |  |
| -> direct |  | -0.701 | -1.158 | -0.186 |
| -> indirect |  | -0.258 | -0.376 | -0.151 |
| -> MBNMA |  | -0.282 | -0.414 | -0.156 |

Table 2.4. Node-splitting analysis of inconsistency (Period)

| Comparison | p-value | Median | 2.50% | 97.50% |
| --- | --- | --- | --- | --- |
| RT_24 vs Placebo_0 | 0.362 |  |  |  |
| -> direct |  | -0.492 | -0.784 | -0.217 |
| -> indirect |  | -0.824 | -1.247 | -0.303 |
| -> MBNMA |  | -0.583 | -0.843 | -0.341 |
| RT_16 vs Placebo_0 | 0.168 |  |  |  |
| -> direct |  | -1.027 | -3.095 | 0.932 |
| -> indirect |  | -0.425 | -0.605 | -0.233 |
| -> MBNMA |  | -0.427 | -0.613 | -0.243 |
| RT_14 vs Placebo_0 | 0.219 |  |  |  |
| -> direct |  | -0.699 | -1.263 | -0.139 |
| -> indirect |  | -0.345 | -0.512 | -0.19 |
| -> MBNMA |  | -0.382 | -0.553 | -0.216 |
| RT_12 vs Placebo_0 | 0.698 |  |  |  |
| -> direct |  | -0.367 | -0.685 | -0.006 |
| -> indirect |  | -0.333 | -0.541 | -0.148 |
| -> MBNMA |  | -0.334 | -0.495 | -0.188 |
| RT_10 vs Placebo_0 | 0.173 |  |  |  |
| -> direct |  | -0.019 | -1.734 | 1.699 |
| -> indirect |  | -0.291 | -0.445 | -0.161 |
| -> MBNMA |  | -0.284 | -0.433 | -0.159 |

Table 2.5. Node-splitting nalysis of inconsistency (Reps)

| Comparison | p-value | Median | 2.50% | 97.50% |
| --- | --- | --- | --- | --- |
| RT_19 vs Placebo_0 | 0.544 |  |  |  |
| -> direct |  | -0.551 | -0.886 | -0.203 |
| -> indirect |  | -0.785 | -1.211 | -0.313 |
| -> MBNMA |  | -0.645 | -0.903 | -0.359 |
|  |  |  |  |  |
| RT_12 vs Placebo_0 | 0.35 |  |  |  |
| -> direct |  | -0.671 | -1.115 | -0.197 |
| -> indirect |  | -0.4 | -0.593 | -0.17 |
| -> MBNMA |  | -0.443 | -0.618 | -0.246 |
|  |  |  |  |  |
| RT_10 vs Placebo_0 | 0.246 |  |  |  |
| -> direct |  | -0.062 | -0.549 | 0.589 |
| -> indirect |  | -0.417 | -0.627 | -0.229 |
| -> MBNMA |  | -0.378 | -0.54 | -0.21 |
|  |  |  |  |  |
| RT_8 vs Placebo_0 | 0.37 |  |  |  |
| -> direct |  | -0.459 | -0.794 | -0.016 |
| -> indirect |  | -0.289 | -0.43 | -0.156 |
| -> MBNMA |  | -0.31 | -0.456 | -0.17 |
|  |  |  |  |  |
| RT_7 vs Placebo_0 | 0.234 |  |  |  |
| -> direct |  | -0.598 | -1.308 | 0.101 |
| -> indirect |  | -0.262 | -0.402 | -0.135 |
| -> MBNMA |  | -0.274 | -0.417 | -0.15 |

Table 2.6. Node-splitting nalysis of inconsistency (Reps/week)

| Comparison | p-value | Median | 2.50% | 97.50% |
| --- | --- | --- | --- | --- |
| RT_2280 vs Placebo_0 | 0.742 |  |  |  |
| -> direct |  | -0.559 | -1.031 | -0.119 |
| -> indirect |  | -0.546 | -0.781 | -0.291 |
| -> MBNMA |  | -0.551 | -0.747 | -0.336 |
| RT_2052 vs Placebo_0 | 0.702 |  |  |  |
| -> direct |  | -0.549 | -1.008 | -0.083 |
| -> indirect |  | -0.555 | -0.775 | -0.287 |
| -> MBNMA |  | -0.549 | -0.744 | -0.336 |
| RT_1080 vs Placebo_0 | 0.399 |  |  |  |
| -> direct |  | -0.717 | -1.145 | -0.284 |
| -> indirect |  | -0.484 | -0.684 | -0.234 |
| -> MBNMA |  | -0.529 | -0.704 | -0.328 |
| RT_840 vs Placebo_0 | 0.038 |  |  |  |
| -> direct |  | 1.044 | -0.362 | 2.454 |
| -> indirect |  | -0.546 | -0.716 | -0.356 |
| -> MBNMA |  | -0.517 | -0.685 | -0.321 |
| RT_420 vs Placebo_0 | 0.483 |  |  |  |
| -> direct |  | -0.331 | -0.728 | 0.057 |
| -> indirect |  | -0.498 | -0.68 | -0.301 |
| -> MBNMA |  | -0.469 | -0.626 | -0.288 |
| RT_360 vs Placebo_0 | 0.222 |  |  |  |
| -> direct |  | -0.699 | -1.096 | -0.279 |
| -> indirect |  | -0.414 | -0.578 | -0.259 |
| -> MBNMA |  | -0.455 | -0.611 | -0.279 |
| RT_200 vs Placebo_0 | 0.327 |  |  |  |
| -> direct |  | -0.196 | -0.634 | 0.26 |
| -> indirect |  | -0.451 | -0.637 | -0.253 |
| -> MBNMA |  | -0.393 | -0.562 | -0.233 |

Table 2.7. Node-splitting nalysis of inconsistency (Sets)

| Comparison | p-value | Median | 2.50% | 97.50% |
| --- | --- | --- | --- | --- |
| RT_4 vs Placebo_0 | 0.191 |  |  |  |
| -> direct |  | -0.511 | -0.744 | -0.238 |
| -> indirect |  | -0.851 | -1.072 | -0.552 |
| -> MBNMA |  | -0.671 | -0.843 | -0.436 |
| RT_3 vs Placebo_0 | 0.104 |  |  |  |
| -> direct |  | -0.709 | -0.935 | -0.488 |
| -> indirect |  | -0.403 | -0.55 | -0.228 |
| -> MBNMA |  | -0.509 | -0.641 | -0.329 |
| RT_2.5 vs Placebo_0 | 0.52 |  |  |  |
| -> direct |  | -0.331 | -0.676 | 0.04 |
| -> indirect |  | -0.435 | -0.559 | -0.261 |
| -> MBNMA |  | -0.427 | -0.539 | -0.276 |
| RT_1 vs Placebo_0 | 0.315 |  |  |  |
| -> direct |  | -0.201 | -0.734 | 0.316 |
| -> indirect |  | -0.17 | -0.233 | -0.1 |
| -> MBNMA |  | -0.174 | -0.222 | -0.112 |

Table 2.7. Node-splitting nalysis of inconsistency (Time)

| Comparison |  | p-value | Median | 2.50% | 97.50% |
| --- | --- | --- | --- | --- | --- |
| RT_60 vs Placebo_0 |  | 0.036 |  |  |  |
| -> direct |  |  | 1.035 | -0.373 | 2.493 |
| -> indirect |  |  | -0.618 | -0.819 | -0.412 |
| -> MBNMA |  |  | -0.582 | -0.782 | -0.353 |
| RT_55 vs Placebo_0 |  | 0.527 |  |  |  |
| -> direct |  |  | -0.601 | -1.223 | 0.056 |
| -> indirect |  |  | -0.539 | -0.744 | -0.279 |
| -> MBNMA |  |  | -0.553 | -0.739 | -0.337 |
| RT_50 vs Placebo_0 |  | 0.441 |  |  |  |
| -> direct |  |  | -0.639 | -0.931 | -0.33 |
| -> indirect |  |  | -0.445 | -0.667 | -0.187 |
| -> MBNMA |  |  | -0.521 | -0.693 | -0.317 |
| RT_45 vs Placebo_0 |  | 0.72 |  |  |  |
| -> direct |  |  | -0.463 | -0.8 | -0.026 |
| -> indirect |  |  | -0.487 | -0.706 | -0.28 |
| -> MBNMA |  |  | -0.489 | -0.646 | -0.295 |
| RT_40 vs Placebo_0 |  | 0.592 |  |  |  |
| -> direct |  |  | -0.374 | -0.753 | -0.1 |
| -> indirect |  |  | -0.48 | -0.652 | -0.255 |
| -> MBNMA |  |  | -0.451 | -0.598 | -0.27 |

### **Supplementary Figures 2.** Node-splitting analysis (density plot).

**Supplementary Figures 2.** Node-splitting analysis (density plot). (A): Frequency; (B): Intensity; (C):Exercises; (D): Period; (E): Reps; (F): Reps/week; (G): Sets; (H) time

# Supplementary file 4: Non-linear functions and models fit comparison

The different doses of physical activity were meta-analysed as independent and unrelated treatments (i.e., “split” NMA). This step is useful to determine which function fits the data better and should subsequently be used in a Model-Based Network Meta-Analysis (MBNMA) (Pedder, 2021). Supplementary Figure 3 shows the different responses (Hedges’ g) of each dose for overall, respectively.

### **Supplementary Figure 3.** “Split” NMA of different Exercise dose.

**Supplementary Figure 3.** “Split” NMA of different Exercise dose. Non-linear functions and models fit comparison. (A): Frequency; (B): Intensity; (C):Exercises; (D): Period; (E): Reps; (F): Reps/week; (G): Sets; (H) time

Supplementary Table 3 shows the fit indices from each of the models fitted. For our data, restricted cubic splines show the best fit and were therefore used in subsequent analyses.

### **Supplementary Table 3.** Models fit comparison

Table 3 shows the fit indices from each of the models fitted. For our data, restricted cubic splines show the best fit and were therefore used in subsequent analyses.

**Table 3.1.** Models fit comparison (Frequency)

| Model | DIC | SD | Deviance | Residual deviance | pD |
| --- | --- | --- | --- | --- | --- |
| Emax  (common treatment effects) | 40.3 | NA | 25.90 | 69.85 | 15.0 |
| Emax  (RANDOM treatment effects) | 9.2 | 0.53 | -15.35 | 28.60 | 25.2 |
| Linear  (common treatment effects) | 40.2 | NA | 25.87 | 69.81 | 15.0 |
| Linear  (random treatment effects) | 9.0 | 0.53 | -15.34 | 28.61 | 24.9 |
| Exponential  (common treatment effects) | 39.8 | NA | 25.48 | 69.42 | 15.0 |
| Exponential  (RANDOM treatment effects) | 9.5 | 0.53 | -15.16 | 28.78 | 25.1 |
| Restricted cubic spline  (common treatment effects; 3 knots) | 3.5 | NA | -11.72 | 32.22 | 15.8 |
| Restricted cubic spline  (random treatment effects; 3 knots) | 5.3 | 0.14 | -14.07 | 29.87 | 19.9 |
| Non-parametric monotonically up  (common treatment effects) | 107.1 | NA | 93.83 | 137.78 | 13.9 |
| Non-parametric monotonically up  (RANDOM treatment effects) | 8.1 | 0.72 | -16.54 | 27.40 | 25.4 |

**Table 3.2.** Models fit comparison (Intensity)

| Model | DIC | SD | Deviance | Residual deviance | pD |
| --- | --- | --- | --- | --- | --- |
| Emax  (common treatment effects) | 39.6 | NA | 26.03 | 71.92 | 14.2 |
| Emax  (RANDOM treatment effects) | 4.0 | 0.59 | -19.22 | 26.67 | 23.8 |
| Linear  (common treatment effects) | 51.7 | NA | 38.29 | 84.18 | 14.0 |
| Linear  (random treatment effects) | 4.6 | 0.62 | -19.33 | 26.56 | 24.4 |
| Exponential  (common treatment effects) | 35.7 | NA | 22.61 | 68.50 | 13.9 |
| Exponential  (RANDOM treatment effects) | 4.1 | 0.55 | -19.03 | 26.86 | 23.7 |
| Restricted cubic spline  (common treatment effects; 3 knots) | 6.1 | NA | -8.26 | 37.63 | 15.1 |
| Restricted cubic spline  (random treatment effects; 3 knots) | 0.9 | 0.21 | -18.42 | 27.47 | 19.8 |
| Non-parametric monotonically up  (common treatment effects) | 107 | NA | 94.55 | 140.44 | 13.1 |
| Non-parametric monotonically up  (RANDOM treatment effects) | 3.5 | 0.95 | -20.51 | 25.38 | 24.6 |

**Table 3.3.** Models fit comparison (Exercises)

| Model | DIC | SD | Deviance | Residual deviance | pD |
| --- | --- | --- | --- | --- | --- |
| Emax  (common treatment effects) | 42.5 | NA | 28.15 | 72.09 | 15.1 |
| Emax  (RANDOM treatment effects) | 9.4 | 0.55 | -15.39 | 28.55 | 25.4 |
| Linear  (common treatment effects) | 42.6 | NA | 28.10 | 72.05 | 15.1 |
| Linear  (random treatment effects) | 9.2 | 0.56 | -15.39 | 28.56 | 25.2 |
| Exponential  (common treatment effects) | 40.1 | NA | 25.72 | 69.67 | 14.9 |
| Exponential  (RANDOM treatment effects) | 8.3 | 0.53 | -15.58 | 28.36 | 24.7 |
| Restricted cubic spline  (common treatment effects; 3 knots) | 12.5 | NA | -2.95 | 40.99 | 16.1 |
| Restricted cubic spline  (random treatment effects; 3 knots) | 6.1 | 0.26 | -15.05 | 28.90 | 21.8 |
| Non-parametric monotonically up  (common treatment effects) | 114.7 | NA | 101.24 | 145.18 | 14.1 |
| Non-parametric monotonically up  (RANDOM treatment effects) | 9.3 | 1.19 | -16.34 | 27.61 | 26.4 |

**Table 3.4.** Models fit comparison (Period)

| Model | DIC | SD | Deviance | Residual deviance | pD |
| --- | --- | --- | --- | --- | --- |
| Emax  (common treatment effects) | 39.9 | NA | 25.36 | 69.30 | 15.2 |
| Emax  (RANDOM treatment effects) | 8.4 | 0.52 | -15.89 | 28.06 | 25.1 |
| Linear  (common treatment effects) | 40.7 | NA | 26.56 | 70.50 | 14.9 |
| Linear  (random treatment effects) | 8.3 | 0.53 | -15.89 | 28.05 | 24.8 |
| Exponential  (common treatment effects) | 40.2 | NA | 25.66 | 69.60 | 15.2 |
| Exponential  (RANDOM treatment effects) | 9.1 | 0.54 | -15.13 | 28.81 | 25.0 |
| Restricted cubic spline  (common treatment effects; 3 knots) | 11.3 | NA | -3.99 | 39.96 | 15.9 |
| Restricted cubic spline  (random treatment effects; 3 knots) | 6.0 | 0.24 | -14.96 | 28.98 | 21.4 |
| Non-parametric monotonically up  (common treatment effects) | 112.6 | NA | 99.37 | 143.31 | 13.8 |
| Non-parametric monotonically up  (RANDOM treatment effects) | 9.2 | 1.04 | -16.29 | 27.65 | 26.2 |

**Table 3.5.** Models fit comparison (Reps)

| Model | DIC | SD | Deviance | Residual deviance | pD |
| --- | --- | --- | --- | --- | --- |
| Emax  (common treatment effects) | 37.7 | NA | 23.95 | 69.84 | 14.5 |
| Emax  (RANDOM treatment effects) | 4.6 | 0.59 | -19.08 | 26.81 | 24.3 |
| Linear  (common treatment effects) | 39.0 | NA | 25.79 | 71.68 | 13.9 |
| Linear  (random treatment effects) | 4.3 | 0.60 | -19.02 | 26.88 | 24.0 |
| Exponential  (common treatment effects) | 35.9 | NA | 22.75 | 68.64 | 13.8 |
| Exponential  (RANDOM treatment effects) | 4.0 | 0.54 | -18.94 | 26.96 | 23.7 |
| Restricted cubic spline  (common treatment effects; 3 knots) | 11.1 | NA | -3.12 | 42.77 | 15.0 |
| Restricted cubic spline  (random treatment effects; 3 knots) | 1.2 | 0.25 | -18.84 | 27.05 | 20.6 |
| Non-parametric monotonically up  (common treatment effects) | 107.9 | NA | 95.35 | 141.24 | 13.2 |
| Non-parametric monotonically up  (RANDOM treatment effects) | 4.0 | 0.98 | -20.36 | 25.53 | 24.9 |

**Table 3.6.** Models fit comparison (Reps/week)

| Model | DIC | SD | Deviance | Residual deviance | pD |
| --- | --- | --- | --- | --- | --- |
| Emax  (common treatment effects) | 31.0 | NA | 18.45 | 65.97 | 13.2 |
| Emax  (RANDOM treatment effects) | -1.0 | 0.57 | -22.21 | 25.31 | 21.9 |
| Linear  (common treatment effects) | 46.8 | NA | 34.57 | 82.09 | 12.9 |
| Linear  (random treatment effects) | -0.9 | 0.70 | -23.01 | 24.51 | 22.8 |
| Exponential  (common treatment effects) | 31.1 | NA | 18.83 | 66.36 | 12.9 |
| Exponential  (RANDOM treatment effects) | -1.0 | 0.56 | -22.51 | 25.02 | 22.2 |
| Restricted cubic spline  (common treatment effects; 3 knots) | 4.4 | NA | -8.92 | 38.603 | 26.5 |
| Restricted cubic spline  (random treatment effects; 3 knots) | -2.3 | 0.24 | -21.28 | 26.24 | 19.5 |
| Non-parametric monotonically up  (common treatment effects) | 30.9 | NA | 18.62 | 66.14 | 13.0 |
| Non-parametric monotonically up  (RANDOM treatment effects) | -0.8 | 1.17 | -23.56 | 23.97 | 23.3 |

**Table 3.7.** Models fit comparison (Sets)

| Model | DIC | SD | Deviance | Residual deviance | pD |
| --- | --- | --- | --- | --- | --- |
| Emax  (common treatment effects) | 31.1 | NA | 18.62 | 66.14 | 13.1 |
| Emax  (RANDOM treatment effects) | -0.7 | 0.59 | -22.26 | 25.27 | 27.2 |
| Linear  (common treatment effects) | 30.8 | NA | 18.57 | 66.09 | 12.8 |
| Linear  (random treatment effects) | -0.6 | 0.59 | -22.26 | 25.26 | 22.4 |
| Exponential  (common treatment effects) | 30.9 | NA | 18.68 | 66.20 | 12.9 |
| Exponential  (RANDOM treatment effects) | -1.7 | 0.57 | -22.61 | 24.91 | 21.8 |
| Restricted cubic spline  (common treatment effects; 3 knots) | -4.6 | NA | -18.03 | 29.49 | 14.2 |
| Restricted cubic spline  (random treatment effects; 3 knots) | -3.4 | 0.13 | -20.39 | 27.13 | 17.5 |
| Non-parametric monotonically up  (common treatment effects) | 102.2 | NA | 90.88 | 138.41 | 12.0 |
| Non-parametric monotonically up  (RANDOM treatment effects) | -0.9 | 0.93 | -23.56 | 23.97 | 23.2 |

**Table 3.8.** Models fit comparison (Time)

| Model | DIC | SD | Deviance | Residual deviance | pD |
| --- | --- | --- | --- | --- | --- |
| Emax  (common treatment effects) | 35.2 | NA | 21.68 | 67.26 | 14.2 |
| Emax  (RANDOM treatment effects) | 4.5 | 0.54 | -18.56 | 27.01 | 23.8 |
| Linear  (common treatment effects) | 35.6 | NA | 22.26 | 67.83 | 14.0 |
| Linear  (random treatment effects) | 4.4 | 0.55 | -18.62 | 26.95 | 23.8 |
| Exponential  (common treatment effects) | 206.9 | NA | 122.382 | 191.600 | 16.0 |
| Exponential  (RANDOM treatment effects) | 4.0 | 0.53 | -18.74 | 26.84 | 23.38 |
| Restricted cubic spline  (common treatment effects; 3 knots) | 6.7 | NA | -7.71 | 37.86 | 15.1 |
| Restricted cubic spline  (random treatment effects; 3 knots) | 2.9 | 0.22 | -17.16 | 28.42 | 20.4 |
| Non-parametric monotonically up  (common treatment effects) | 34.9 | NA | 21.52 | 67.10 | 14.1 |
| Non-parametric monotonically up  (RANDOM treatment effects) | 3.9 | 0.91 | -20.02 | 25.55 | 24.6 |

*Note.* DIC = Deviance Information Criterion; SD = Between-study Standard Deviation; pD: Number of estimated parameters; NA = Not Applicable. The SD is presented as the main value and (95% Credible Intervals).

Further to model fit indices, deviance plots showing the contribution of each data point to the residual deviance are also useful to confirm the robustness of model selection (Pedder H, 2021). Each data point should contribute about 1 to the posterior mean deviance, which indicates good model fit (Dias S, et al., 2013). The deviance plot for treatment effects (Supplementary File 4) confirm the robustness of our model selection.

### **Supplementary Figure 4**. The deviance plot for treatment effects.

**Supplementary Figure 4**. The deviance plot for treatment effects. (A): Frequency; (B): Intensity; (C):Exercises; (D): Period; (E): Reps; (F): Reps/week; (G): Sets; (H) time

# Supplementary file 5: Characteristics of included studies

This supplementary file provides the datasets (i.e., at global exercise and agent levels) used in this study. The *studyID* indicates the author’s name 、the year of publication and study number. The *agent* indicates groups in studies. The *y* indicates the average of all values of research variable. The *n* indicates the number of participants in the study.The *se* indicates the standard error of the mean. The *sd_session* indicates the standard deviation of the mean. The *frequency* is the number of days per week that participants were participated in physical activity.The *Period* indicates exercise period in the study. The *Set* indicates the group of doses by approximation. The *Reps* indicates the times of repetition by approximation. The *Intensity* indicates the percentage of participants completed OneRepMax. The *Reps/week* indicates the number of weekly repetitions by approximation. The *Exercises* indicates the number of exercises per set that participants were involved in physical activity. The *Time* indicates the duration of each training session.

| **studyID** | **agent** | **y** | **n** | **se** | **sd** | **Frequency** | **Period** | **Set** | **Reps** | **Intensity** | **Reps/week** | **Exercises** | **Time** |
| --- | --- | --- | --- | --- | --- | --- | --- | --- | --- | --- | --- | --- | --- |
| Study ID | CON | 9.2 | 6 | 0.86 | 2.1 | 0 | 0 | 0 | 0 | 0 | 0 | 0 | 0 |
|  | RT | 8.1 | 6 | 0.49 | 1.2 | 3 | 16 | 3.5 | 9.5 | 75 | 598.5 | 6 | 40 |
| Soumitra Mondal et al. (2021)^1^ | CON | 8.22 | 31 | 0.17 | 0.97 | 0 | 0 | 0 | 0 | 0 | 0 | 0 | 0 |
|  | RT | 7.73 | 32 | 0.16 | 0.88 | 3 | 12 | 4 | 19 | 45 | 2052 | 9 | 40 |
| Xiaojun Ma et al. (2024)^2^ | CON | 8.17 | 31 | 0.17 | 0.97 | 0 | 0 | 0 | 0 | 0 | 0 | 0 | 0 |
|  | RT | 7.57 | 32 | 0.16 | 0.89 | 3 | 24 | 4 | 19 | 45 | 2052 | 9 | 40 |
| Xiaojun Ma et al. (2024)^3^ | CON | 7.95 | 33 | 0.16 | 0.92 | 0 | 0 | 0 | 0 | 0 | 0 | 0 | 0 |
|  | RT | 7.37 | 31 | 0.15 | 0.85 | 3 | 12 | 3 | 12 | 50 | 1080 | 10 | 50 |
| Xiaojun Ma et al. (2024)^4^ | CON | 7.95 | 33 | 0.16 | 0.92 | 0 | 0 | 0 | 0 | 0 | 0 | 0 | 0 |
|  | RT | 7.53 | 34 | 0.16 | 0.93 | 3 | 12 | 4 | 19 | 25 | 2280 | 10 | 50 |
| Xiaojun Ma et al. (2024)^5^ | CON | 7.93 | 33 | 0.13 | 0.75 | 0 | 0 | 0 | 0 | 0 | 0 | 0 | 0 |
|  | RT | 7.11 | 31 | 0.13 | 0.75 | 3 | 24 | 3 | 12 | 50 | 1080 | 10 | 50 |
| Xiaojun Ma et al. (2024)^6^ | CON | 7.93 | 33 | 0.13 | 0.75 | 0 | 0 | 0 | 0 | 0 | 0 | 0 | 0 |
|  | RT | 7.24 | 34 | 0.15 | 0.85 | 3 | 24 | 4 | 19 | 25 | 2280 | 10 | 50 |
| Xiaojun Ma et al. (2024)^7^ | CON | 7.7 | 24 | 0.12 | 0.6 | 0 | 0 | 0 | 0 | 0 | 0 | 0 | 0 |
|  | RT | 7.1 | 23 | 0.19 | 0.9 | 3 | 12 | 2.5 | 7 | 70 | 420 | 8 | 55 |
| Nikolaos P. E. Kadoglou et al. (2020) | CON | 7.2 | 28 | 0.08 | 0.4 | 0 | 0 | 0 | 0 | 0 | 0 | 0 | 0 |
|  | RT | 7 | 29 | 0.07 | 0.4 | 2 | 24 | 1 | 10 | 70 | 200 | 10 | 40 |
| Jürgen Giessing et al. (2022) | CON | 8.3 | 31 | 0.09 | 0.5 | 0 | 0 | 0 | 0 | 0 | 0 | 0 | 0 |
|  | RT | 7.6 | 31 | 0.04 | 0.2 | 3 | 14 | 3 | 8 | 70 | 360 | 5 | 45 |
| CARMEN CASTANEDA et al.(2002) | CON | 8.4 | 9 | 0.6 | 1.8 | 0 | 0 | 0 | 0 | 0 | 0 | 0 | 0 |
|  | RT | 8.4 | 9 | 0.6 | 1.8 | 3 | 10 | 2 | 12 | 70 | 720 | 10 | 40 |
| J. C. Baldi et al.(2003) | CON | 7.33 | 63 | 0.19 | 1.49 | 0 | 0 | 0 | 0 | 0 | 0 | 0 | 0 |
|  | RT | 7.35 | 64 | 0.2 | 1.57 | 3 | 12 | 2.5 | 8 | 80 | 420 | 7 | 45 |
| Ronald J. Sigal et al.(2016) | CON | 7.51 | 63 | 0.19 | 1.47 | 0 | 0 | 0 | 0 | 0 | 0 | 0 | 0 |
|  | RT | 7.18 | 64 | 0.19 | 1.52 | 3 | 24 | 2.5 | 8 | 80 | 420 | 7 | 45 |
| N.W.Cheung et al.(2009) | CON | 7.17 | 12 | 0.2 | 0.7 | 0 | 0 | 0 | 0 | 0 | 0 | 0 | 0 |
|  | RT | 8.24 | 10 | 0.67 | 2.13 | 3 | 12 | 4 | 10 | 50 | 840 | 7 | 60 |
| Vanessa Neves de Oliveira et al.(2011) | CON | 7.4 | 63 | 0.13 | 1 | 0 | 0 | 0 | 0 | 0 | 0 | 0 | 0 |
|  | RT | 7.2 | 64 | 0.2 | 1.6 | 3 | 12 | 4 | 10 | 50 | 840 | 7 | 60 |

# List of included studies

1. Soumitra Mondal, Muluken Gebeyehu, Kesatie Legesse, Mulay Gebretensay. Effects of Aerobic and Resistance Exercises on Selected Physiological Biochemical and Anthropometric Variables among Type 2 Diabetic Patients in Dilla, Ethiopia. Indian Journal of Public Health Research & Development. 2021.
2. Ma X, Lin X, Zhou L, Li W, Yi Q, Lei F, Tang X, Ai Y, Zhan Y, Luo H, Wang L, Lei F, He B, Yang F, Ruan S. The effect of blood flow-restrictive resistance training on the risk of atherosclerotic cardiovascular disease in middle-aged patients with type 2 diabetes: a randomized controlled trial. Front Endocrinol (Lausanne). 2024 Oct 1;15:1482985. doi: 10.3389/fendo.2024.1482985..
3. Ma X, Ai Y, Lei F, Tang X, Li Q, Huang Y, Zhan Y, Mao Q, Wang L, Lei F, Yi Q, Yang F, Yin X, He B, Zhou L, Ruan S. Effect of blood flow-restrictive resistance training on metabolic disorder and body composition in older adults with type 2 diabetes: a randomized controlled study. Front Endocrinol (Lausanne). 2024 Jun 6;15:1409267. doi: 10.3389/fendo.2024.1409267..
4. Kadoglou NP, Fotiadis G, Athanasiadou Z, Vitta I, Lampropoulos S, Vrabas IS. The effects of resistance training on ApoB/ApoA-I ratio, Lp(a) and inflammatory markers in patients with type 2 diabetes. Endocrine. 2012 Dec;42(3):561-9. doi: 10.1007/s12020-012-9650-y..
5. Giessing J, Eichmann B, Kemmler W, Westcott WL, Winett R, Busuttil K, Steele J, Fisher JP. The effects of adding high-intensity of effort resistance training to routine care in persons with type II diabetes: An exploratory randomized parallel-group time-series study. Physiol Behav. 2022 Mar 1;245:113677. doi: 10.1016/j.physbeh.2021.113677. Epub 2021 Dec 15. PMID: 34921836..
6. Castaneda C, Layne JE, Munoz-Orians L, Gordon PL, Walsmith J, Foldvari M, Roubenoff R, Tucker KL, Nelson ME. A randomized controlled trial of resistance exercise training to improve glycemic control in older adults with type 2 diabetes. Diabetes Care. 2002 Dec;25(12):2335-41. doi: 10.2337/diacare.25.12.2335..
7. Baldi JC, Snowling N. Resistance training improves glycaemic control in obese type 2 diabetic men. Int J Sports Med. 2003 Aug;24(6):419-23. doi: 10.1055/s-2003-41173.
8. Cheung NW, Cinnadaio N, Russo M, Marek S. A pilot randomised controlled trial of resistance exercise bands in the management of sedentary subjects with type 2 diabetes. Diabetes Res Clin Pract. 2009 Mar;83(3):e68-71. doi: 10.1016/j.diabres.
9. Sigal RJ, Kenny GP, Boulé NG, Wells GA, Prud'homme D, Fortier M, Reid RD, Tulloch H, Coyle D, Phillips P, Jennings A, Jaffey J. Effects of aerobic training, resistance training, or both on glycemic control in type 2 diabetes: a randomized trial. Ann Intern Med. 2007 Sep 18;147(6):357-69. doi: 10.7326/0003-4819-147-6-200709180-00005. PMID: 17876019..
10. de Oliveira VN, Bessa A, Jorge ML, Oliveira RJ, de Mello MT, De Agostini GG, Jorge PT, Espindola FS. The effect of different training programs on antioxidant status, oxidative stress, and metabolic control in type 2 diabetes. Appl Physiol Nutr Metab. 2012 Apr;37(2):334-44. doi: 10.1139/h2012-004....

# References

1	[ter Veer E, van Oijen MGH, van Laarhoven HWM. The Use of (Network) Meta-Analysis in Clinical Oncology. *Front Oncol* 2019;**9**:822. doi:](http://paperpile.com/b/b8T1z2/YP82p)[10.3389/fonc.2019.00822](http://dx.doi.org/10.3389/fonc.2019.00822)

2	[Wheeler DC, Hickson DA, Waller LA. Assessing local model adequacy in Bayesian hierarchical models using the partitioned deviance information criterion. Computational Statistics & Data Analysis. 2010;**54**:1657–71. doi:](http://paperpile.com/b/b8T1z2/jIaJT)[10.1016/j.csda.2010.01.025](http://dx.doi.org/10.1016/j.csda.2010.01.025)

3	Pedder H. MBNMAdose: An R package for incorporating dose-response information into Network Meta-Analysis. In: Evidence Synthesis and Meta-Analysis in R Conference 2021. 2021. <https://research-information.bris.ac.uk/en/publications/mbnmadose-an-r-package-for-incorporating-dose-response-information>

4	[Dias S, Sutton AJ, Ades AE, *et al.* Evidence synthesis for decision making 2: a generalized linear modeling framework for pairwise and network meta-analysis of randomized controlled trials. *Med Decis Making* 2013;**33**:607–17. doi:](http://paperpile.com/b/b8T1z2/6GsS)[10.1177/0272989X12458724](http://dx.doi.org/10.1177/0272989X12458724)
